# VKORC1L1–mediated vitamin K recycling counters ferroptosis to promote endothelial repair

**DOI:** 10.1038/s41598-026-54463-7

**Published:** 2026-06-19

**Authors:** Elena Repges, Adem Aksoy, Katrin J. Czogalla-Nitsche, Andreas Zietzer, Cornelius Müller, Johannes Oldenburg, Sebastian Zimmer, Georg Nickenig, Vedat Tiyerili, Muntadher Al Zaidi

**Affiliations:** 1 Department of Internal Medicine II, Heart Center Bonn, University Hospital, Bonn, Germany; 2 Institute for Experimental Hematology and Transfusion Medicine, University Hospital Bonn, Bonn, Germany; 3 Department of Internal Medicine II, Heart Center Bonn, University Hospital Bonn, Venusberg-Campus 1, 53127 Bonn, Germany

**Keywords:** Endothelium, Ferroptosis, Lipid peroxidation, Vitamin K, VKORC1L1, Biochemistry, Cell biology, Diseases, Molecular biology

## Abstract

Vitamin K is classically known for hepatic γ-carboxylation via VKORC1, yet accumulating evidence suggests broader redox-regulatory roles. Here, we characterize the VKORC1 paralogue VKORC1L1 as an anti-oxidative enzyme that couples the vitamin K cycle to ferroptosis defense in the endothelium. In human coronary artery endothelial cells (HCAEC), menaquinone-7 (Vitamin K2, MK-7) increased viability and attenuated RSL3-induced lipid peroxidation, ferroptosis, and NF-κB-dependent inflammatory activation. In vivo, a MK-7 enriched diet accelerated reendothelialization after electric carotid injury in C57BL/6J mice without altering hemostasis. Silencing VKORC1L1, but not the coagulation-linked VKORC1, diminished HCAEC proliferation, raised reactive oxygen species, and triggered NF-κB activation. Importantly, MK-7 failed to rescue these effects in VKORC1L1-deficient cells, supporting a functional requirement for VKORC1L1 in vitamin K-mediated cytoprotection. Transcriptomics of VKORC1L1-deficient cells revealed a lipid peroxide/NF-κB/TNF pathway that amplified oxidative inflammation. This signaling axis was blocked by the lipid-radical scavenger ferrostatin-1 or by inhibiting TNF shedding. Collectively, these data identify VKORC1L1 as a redox-responsive component of the endothelial vitamin K cycle that limits lipid peroxides, suppresses ferroptosis, restrains TNF-driven inflammation, and promotes vascular repair.

**Supplementary Information:**

The online version contains supplementary material available at 10.1038/s41598-026-54463-7.

## Introduction

Endothelial cells line the inner surface of blood vessels and orchestrate vascular tone, barrier integrity, and hemostasis. Because of this sentinel position, they are persistently exposed to mechanical, metabolic, and inflammatory stressors that impair function and precipitate cardiovascular disease^1^. Endothelial dysfunction, marked by reduced nitric-oxide bioavailability, pro-inflammatory activation, diminished angiogenic capacity, endothelial-to-mesenchymal-transition (EndMT), and ultimately cell death, constitutes the earliest detectable change in atherosclerotic lesions and drives cardiovascular disease progression^1,2^.

Vitamin K is a highly lipophilic quinone that mainly exists as phylloquinone (K1) in green plants and menaquinones (K2, MK-n) in animal products and fermented foods^3,4^. Among these, menaquinone-7 (MK-7) exhibits the highest oral bioavailability^5^.

Vitamin K-hydroquinone serves as an obligate co-factor for γ-carboxylation of vitamin K-dependent (VKD) proteins including the hepatic coagulation factors II, VII, IX, and X. However, there is also a plethora of extrahepatic VKD-proteins including proteins implicated in inhibiting ectopic calcification (e.g. Matrix-Gla-protein), preserving vascular integrity, and regulating immunity^3,6^. Beyond its role in γ-carboxylation, vitamin K-hydroquinone is a potent radical-trapping antioxidant that quenches lipid peroxidation and alleviates glutathione-depletion-induced oxidative stress^7–9^.

Regeneration of vitamin K hydroquinone from its 2,3-epoxide in the endoplasmic reticulum (ER) is catalyzed by two paralogous enzymes: VKORC1 (vitamin-K-2,3-epoxide-reductase-complex-subunit-1), the canonical target of vitamin K antagonists, and VKORC1L1 (VKORC1-like-1). VKORC1 is liver-enriched and indispensable for coagulation^10^. In contrast, VKORC1L1 is dispensable for hemostasis^11^ but has been linked to intracellular anti-oxidation^12^, vascular smooth muscle cell function^13^, and protection against ferroptosis^14^, a lipid-peroxide-driven form of regulated cell death implicated in cardiovascular diseases^15–18^. Despite these insights, our current understanding of VKORC1L1 is limited and there is no experimental data regarding the role of VKORC1L1 in endothelial biology.

Most work on vitamin K in the vasculature has focused on preventing calcification, a feature of advanced vascular disease, while its additional functions in vascular and endothelial homeostasis remain less understood. Previous studies indicate that vitamin K can improve endothelial function by modulating nitric oxide production, endothelium-dependent vasodilation, and cellular senescence^19,20^. However, whether vitamin K actively supports endothelial regeneration after injury, and which cellular factors mediate such an effect, is unknown.

Here we test the hypothesis that vitamin K preserves endothelial integrity by limiting lipid peroxidation and ferroptosis via VKORC1L1. We combine loss-of-function genetics, ferroptosis assays, and a murine carotid-injury model to determine how VKORC1L1 modulates lipid peroxidation, inflammatory activation, and endothelial repair.

## Results

### Vitamin K2 (MK-7) promotes a pro-regenerative endothelial phenotype

**Fig. 1:**
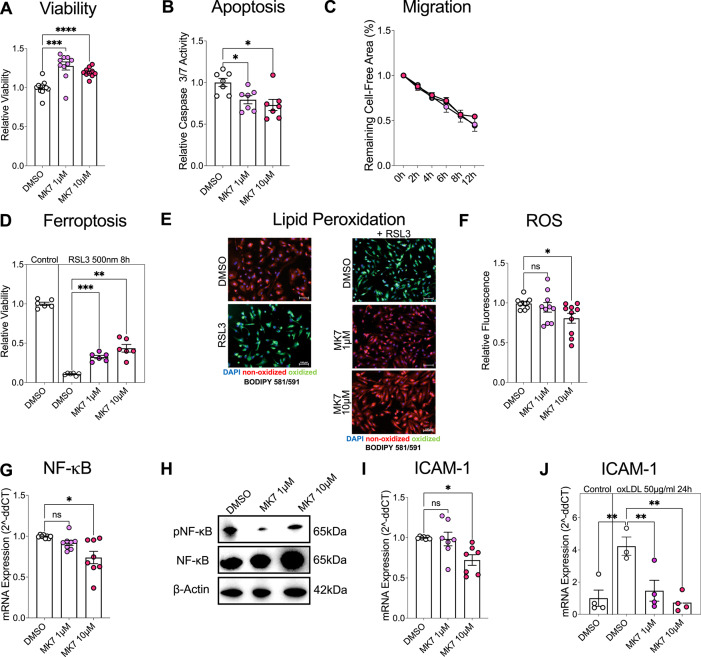
**Vitamin K2 (MK-7) promotes an antioxidant**,** pro-regenerative endothelial phenotype**. **A–B **Cell viability (AlamarBlue) and apoptosis (caspase-3/7) in HCAEC after 24 h MK-7 (1–10 µM); *n* = 10–12 (A) and 7 (B). **C **Scratch-wound migration over 12 h; *n* = 4. **D **Viability after ferroptosis induction with RSL3 (500 nM, 8 h) ± MK-7; *n* = 6. **E **Lipid peroxidation (BODIPY 581/591 C11) by fluorescence microscopy (left) and flow cytometry (right) under the treatments in D. *n* = 3. **F **Total ROS (DCFDA) following 24 h MK-7 and a 1 h H₂O₂ challenge (75 µM); *n* = 10. **G** qPCR for NF-κB after MK-7 treatment in LPS-stimulated cells; *n* = 8. **H** Representative immunoblot of NF-κB and phosphorylated NF-κB with β-actin loading control, under the treatments in G. **I-J** qPCR of ICAM-1 under resting conditions and oxLDL stimulation ± MK-7; *n* = 7 (I) and 4 (J). Statistics: one-way ANOVA + Dunnett (A-B, D, F-G, I-J); two-way ANOVA (C). Data are mean ± SEM. *P < 0.05; **P < 0.01; ***P < 0.001; ****P < 0.0001.

To determine how vitamin K2 influences endothelial function, primary human coronary artery endothelial cells (HCAEC) were exposed to menaquinone-7 (MK-7), the most bioavailable vitamin K vitamer^5^. MK-7 increased HCAEC viability with significant gains of 27 % at 1 µM and 20 % at 10 µM (Fig. 1A). Concordantly, caspase 3/7 activity as a surrogate of apoptosis fell by 21 % and 27 % at 1 µM and 10µM, respectively (Fig. 1B), while scratch assays showed no change in basal migration (Fig. 1C).

Because lipid-peroxidation-driven ferroptosis is implicated in vascular injury and can be suppressed by vitamin K^21^, we next challenged HCAEC with the GPX4 inhibitor RSL3 (8 h, 500 nM). RSL3 reduced cell viability to 10 ± 2 % of control, but co-treatment with 1 µM or 10 µM MK-7 partially restored cell viability to 32 ± 5 % and 44 ± 11% respectively (Fig. 1D). BODIPY 581/591 C11 staining confirmed that MK-7 curtailed RSL3-induced lipid peroxide accumulation (Fig. 1E). As excessive ROS formation contributes to lipid peroxide generation^15^, we measured intracellular ROS levels following H₂O₂ challenge and observed that MK-7 reduced ROS levels at 10 µM (Fig. 1F). Specificity of the DCFDA-based ROS assay was confirmed using H₂O₂ as a positive control and catalase as a negative control (Figure S1).

In endothelial cells, ROS-dependent NF-κB activation drives the upregulation of adhesion molecules resulting in leukocyte adhesion and diapedesis^22,23^. Correspondingly, MK-7 treatment reduced NF-κB expression and activation at both the mRNA and protein level (Fig. 1G and H), as well as ICAM-1 expression under both basal conditions and following pro-inflammatory oxLDL stimulation (Fig. 1I and J).

Collectively, these findings show that MK-7 enhances endothelial viability, suppresses ferroptosis-associated lipid peroxidation, and attenuates NF-κB-dependent inflammatory activation, consistent with a pro-regenerative endothelial phenotype.

**A–B **Cell viability (AlamarBlue) and apoptosis (caspase-3/7) in HCAEC after 24 h MK-7 (1–10 µM); *n* = 10–12 (A) and 7 (B). **C **Scratch-wound migration over 12 h; *n* = 4. **D **Viability after ferroptosis induction with RSL3 (500 nM, 8 h) ± MK-7; *n* = 6. **E **Lipid peroxidation (BODIPY 581/591 C11) by fluorescence microscopy (left) and flow cytometry (right) under the treatments in D. *n* = 3. **F **Total ROS (DCFDA) following 24 h MK-7 and a 1 h H₂O₂ challenge (75 µM); *n* = 10. **G** qPCR for NF-κB after MK-7 treatment in LPS-stimulated cells; *n* = 8. **H** Representative immunoblot of NF-κB and phosphorylated NF-κB with β-actin loading control, under the treatments in G. **I-J** qPCR of ICAM-1 under resting conditions and oxLDL stimulation ± MK-7; *n* = 7 (I) and 4 (J). Statistics: one-way ANOVA + Dunnett´s (A-B, D, F-G, I-J); two-way ANOVA (C). Data are mean ± SEM. *P < 0.05; **P < 0.01; ***P < 0.001; ****P < 0.0001.

## MK-7-promoted endothelial cell regeneration is partly shared by Vitamin K1

Menaquinones (K2) exhibit greater oral bioavailability than phylloquinone (K1)^5^, yet many of their cellular actions overlap^20,21^. To test whether MK-7´s pro-regenerative effects are unique to K2 vitamers, we repeated the key assay with K1. Vitamin K1 did not significantly alter basal endothelial cell viability (Figure S2A) or apoptosis (Figure S2B). However, co-incubation with K1 suppressed RSL3-induced ferroptosis (Figure S2C), closely matching the protection conferred by MK-7. Thus, although K1 is less efficiently absorbed in vivo and does not influence baseline viability or apoptosis, it retains the capacity to protect HCAEC from ferroptosis in vitro, indicating that ferroptosis defense in the endothelium is a shared property of vitamin K forms.

## Vitamin K2 (MK-7) accelerates endothelial regeneration in mice

**Fig. 2:**
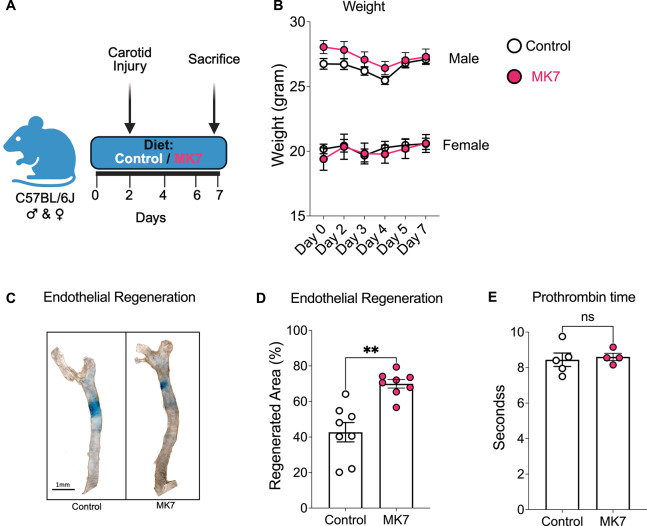
Vitamin K2 (MK-7) accelerated endothelial regeneration in mice. **A **Study design: female and male C57BL/6J mice received MK-7-enriched chow (100 µg g⁻¹) or control diet beginning 2 days before electric denudation of the left common carotid artery. Sacrifice was performed 5 days after injury. **B **Body-weight trajectory; *n* = 8 per group (4 ♂/4 ♀). **C**,** D **Reendothelialization quantified by Evans-Blue staining on en-face carotid preparations; scale bar 1 mm; *n* = 8. **E **Plasma prothrombin time at sacrifice; *n* = 4–5. Statistics: two-tailed unpaired t-test (C, E). Values are mean ± SEM; *P < 0.05, **P < 0.01.

Cardiovascular diseases often develop upon injury of the endothelial monolayer. In vivo, vascular health is thus dependent on the regenerating capacity of the endothelium^1^. To translate our in vitro findings, we tested whether MK-7 promotes endothelial repair in a murine denudation model. Female and male C57BL/6J mice were randomized to a control chow or an MK-7-enriched diet (100 µg/g) beginning two days before surgery (Fig. 2A). Body-weight curves did not differ between groups (Fig. 2B). Five days after electric denudation of the left common carotid artery, Evans Blue was injected intravenously, and *en-face* imaging of explanted carotid arteries was performed. MK-7 increased the reendothelization from 42.7 ± 15 % in controls to 70.0 ± 7 % in the MK-7 group, confirming a pro-regenerative effect in vivo (Fig. 2C and D). Prothrombin time remained unchanged (Fig. 2E), indicating that the MK-7 dose did not create a hypercoagulable state. Thus, dietary MK-7 enhances endothelial regeneration after arterial injury, consistent with its protective activity observed in cultured HCAEC.

**A **Study design: female and male C57BL/6J mice received MK-7-enriched chow (100 µg g⁻¹) or control diet beginning 2 days before electric denudation of the left common carotid artery. Sacrifice was performed 5 days after injury. **B **Body-weight trajectory; *n* = 8 per group (4 ♂/4 ♀). **C**,** D **Reendothelialization quantified by Evans-Blue staining on en-face carotid preparations; scale bar 1 mm; *n* = 8. **E **Plasma prothrombin time at sacrifice; *n* = 4–5. Statistics: two-tailed unpaired t-test (C, E). Values are mean ± SEM; *P < 0.05, **P < 0.01.

## MK-7 and vitamin K1 blunt endothelial-to-mesenchymal transition (EndMT)

Persistent vascular oxidative stress promotes adverse cellular remodeling including TGF-ß (transforming growth factor beta)-mediated EndMT, a major hallmark of unstable atherosclerotic plaques^24,25^. Given the fact that vitamin K acts as a potent antioxidant and restored endothelial viability, we asked whether it also limits EndMT. HCAEC were exposed for 96 h to TGF-β2 and interleukin-1β. The cells adopted a spindle-shaped morphology (Figure S3A) and up-regulated the mesenchymal markers SM22, Vimentin and N-Cadherin (Figure S3B – S3E), while endothelial eNOS, CD31, vWF and VE-Cadherin were lost (Figure S3F – S3J). Co-incubation with MK-7, and to a similar extent vitamin K1 (Figure S4A – S4B), blunted these phenotypic shifts: Mesenchymal protein induction fell (Figure S3B – S3E) while endothelial marker proteins were restored (Figure S3F – S3J). EndMT also decreased VKORC1L1 mRNA and protein levels, with a smaller reduction observed for VKORC1 (Figure S3K – S3M). Together, these observations provide further evidence that vitamin K preserves endothelial phenotypes under inflammatory-oxidative conditions.

## Silencing VKORC1L1 impairs endothelial regeneration and heightens oxidative-inflammatory stress

**Fig. 3:**
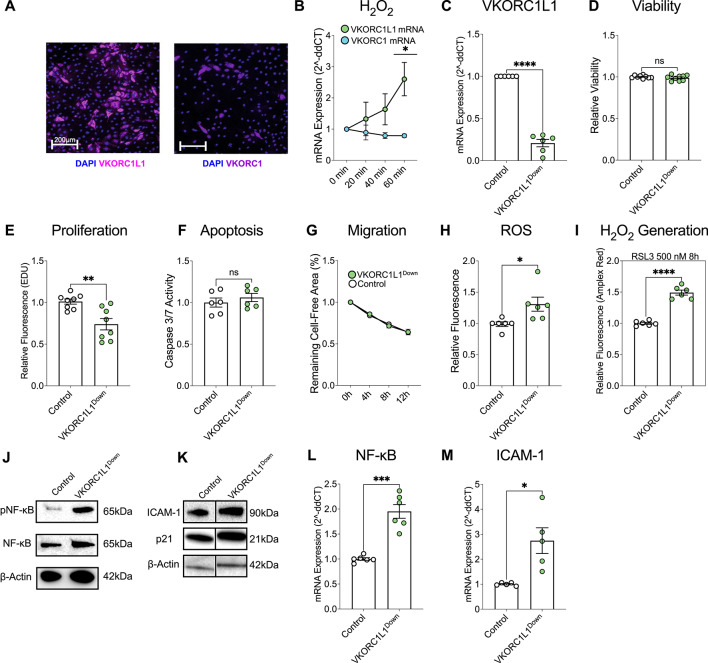
VKORC1L1 silencing impairs endothelial regeneration and heightens oxidative-inflammatory stress. **A **Endogenous VKORC1L1 (left) and VKORC1 (right) immunofluorescence in resting HCAEC; scale = 200 μm (*n* = 3). **B **Time-course qPCR following H₂O₂ treatment (75 µM) shows selective VKORC1L1 up-regulation (*n* = 5). **C** qPCR validation of VKORC1L1 knockdown 24 h after siRNA transfection (*n* = 6). **D–G **Effects of VKORC1L1 silencing on viability (AlamarBlue, *n* = 9), proliferation (EdU, *n* = 8), apoptosis (caspase-3/7, *n* = 6), and migration (scratch assay, *n* = 6). **H–I **ROS levels (DCFDA, *n* = 6, H) and RSL3-stimulated H₂O₂ release (Amplex Red, *n* = 6, I) after VKORC1L1 knockdown. **J **Representative immunoblot of phosphorylated NF-κB and NF-κB (*n* = 2) with β-actin loading control. **K** Representative immunoblot of ICAM-1 (*n* = 3) and p21 (*n* = 3) with β-actin loading control. **L-M **qPCR of NF-κB and ICAM-1 expression following VKORC1L1 silencing (*n* = 6 (L) and 5(M)). Statistics: two-way ANOVA (B, G) or unpaired two-tailed t-test (C-F, H, I, L, M). Data are mean ± SEM; *P < 0.05, **P < 0.01, ***P < 0.001, ****P < 0.0001.

The radical-trapping vitamin K-hydroquinone is regenerated from its 2,3-epoxide by the ER enzymes VKORC1 and VKORC1L1. VKORC1 sustains hepatic γ-carboxylation and VKORC1^−/−^ mice succumb to early postnatal bleeding^26,27^, whereas VKORC1L1 has been implicated in intracellular anti-oxidation and ferroptosis suppression^12,14^. However, its role in the endothelium remains unknown. Interrogation of GTEx and Human Protein Atlas datasets confirmed that VKOR enzymes are present in healthy arterial tissue and vascular endothelial cells^28^. Proteomic data from human coronary atheroma revealed selective upregulation of VKORC1L1, but not VKORC1^29^. In resting HCAEC, immunofluorescence demonstrated expression of both VKOR proteins with VKORC1L1 exhibiting higher fluorescence intensity (Fig. 3A). Exposure to 75 µM H_2_O_2_, a physiologically relevant oxidant in the vasculature^30^, induced a time-dependent rise in VKORC1L1 mRNA whereas VKORC1 transcript levels remained unchanged (Fig. 3B). These findings identify VKORC1L1 as the oxidant-responsive VKOR isoform in HCAEC.

Next, VKORC1L1 was silenced using siRNA, achieving efficient knockdown (Fig. 3C). VKORC1L1 silencing did not modulate basal viability, apoptosis, or migration (Fig. 3D, F and G). However, it led to reduced cell proliferation (Fig. 3E). Moreover, VKORC1L1-knockdown promoted a rise in ROS formation (Fig. 3H) and VKORC1L1-deficient cells generated more H_2_O_2_ (Amplex Red) during RSL3-induced ferroptosis (Fig. 3I).

The increase in ROS following VKORC1L1 silencing was accompanied by enhanced NF-κB expression and activation (Fig. 3J, L), as well as increased expression of the adhesion molecules ICAM-1 (Fig. 3K, M) and VCAM-1 (Figure S5A). In addition, IL-6 secretion increased significantly, further supporting a pro-inflammatory endothelial phenotype (Figure S5B). Expression of the stress-responsive cell-cycle inhibitor p21 was also increased following VKORC1L1 silencing (Fig. 3K), consistent with the observed reduction in endothelial proliferation. Collectively, these findings support a role for VKORC1L1 as a redox-responsive vitamin K reductase in the endothelium whose loss promotes oxidative stress, inflammatory activation, and impaired endothelial proliferative capacity.

**A **Endogenous VKORC1L1 (left) and VKORC1 (right) immunofluorescence in resting HCAEC; scale = 200 μm (*n* = 3). **B **Time-course qPCR following H₂O₂ treatment (75 µM) shows selective VKORC1L1 up-regulation (*n* = 5). **C** qPCR validation of VKORC1L1 knockdown 24 h after siRNA transfection (*n* = 6). **D–G **Effects of VKORC1L1 silencing on viability (AlamarBlue, *n* = 9), proliferation (EdU, *n* = 8), apoptosis (caspase-3/7, *n* = 6), and migration (scratch assay, *n* = 6). **H–I **ROS levels (DCFDA, *n* = 6, H) and RSL3-stimulated H₂O₂ release (Amplex Red, *n* = 6, I) after VKORC1L1 knockdown. **J **Representative immunoblot of phosphorylated NF-κB and NF-κB (*n* = 2) with β-actin loading control. **K** Representative immunoblot of ICAM-1 (*n* = 3) and p21 (*n* = 3) with β-actin loading control. **L-M **qPCR of NF-κB and ICAM-1 expression following VKORC1L1 silencing (*n* = 6 (L) and 5(M)). Statistics: two-way ANOVA (B, G) or unpaired two-tailed t-test (C-F, H, I, L, M). Data are mean ± SEM; *P < 0.05, **P < 0.01, ***P < 0.001, ****P < 0.0001.

### VKORC1-silencing elicits a pro-regenerative, anti-oxidative phenotype and is accompanied by VKORC1L1 upregulation

**Fig. 4:**
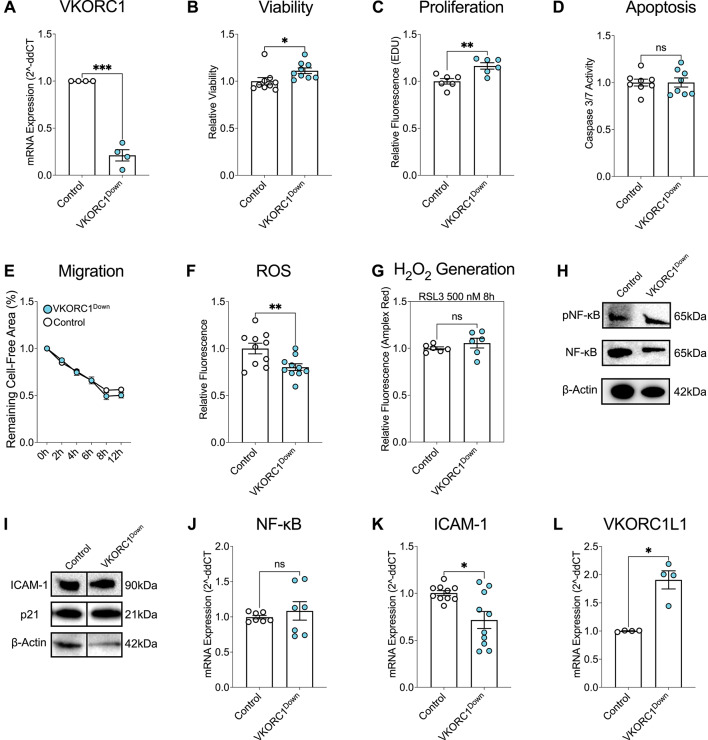
**VKORC1-silencing elicits a pro-regenerative**,** anti-oxidative phenotype and is accompanied by VKORC1L1 upregulation**. **A** qPCR validation of VKORC1 knock-down 24 h after siRNA transfection (*n* = 6). **B–D **AlamarBlue viability (*n* = 9), EdU proliferation (*n* = 6), and caspase-3/7 activity (*n* = 8) in HCAEC after VKORC1 silencing. **E **Scratch-wound migration over 12 h (*n* = 6). **F–G **Total ROS (DCFDA, *n* = 10, F) and RSL3-stimulated H₂O₂ release (Amplex Red, *n* = 6, G) following VKORC1 silencing. **H** Representative immunoblot of phosphorylated NF-κB and NF-κB after VKORC1 knockdown (*n* = 2) with β-actin loading control. **I** Representative immunoblot of p21 and ICAM-1 (*n* = 3) with β-actin loading control. **J**,** K**,** L** qPCR for NF-κB (*n* = 7), ICAM-1 (*n* = 10), and VKORC1L1 (*n* = 4) in VKORC1-deficient cells. Statistics: unpaired two-tailed t-test (A–D, F, G, J, K, L) or two-way ANOVA (E). Values are mean ± SEM; *P < 0.05, **P < 0.01.

Silencing the canonical reductase VKORC1 (Fig. 4A) resulted in a phenotype opposite to that observed after VKORC1L1 knockdown. Viability and proliferation increased, whereas apoptosis and migration remained unaltered (Fig. 4B and E). Total ROS levels fell significantly, while H_2_O_2_ production remained unchanged (Fig. 4F and G). Moreover, VKORC1 knockdown did not alter NF-κB expression or activation (Fig. 4H and J), nor did it modulate VCAM-1 expression (Figure S5C). Notably, VKORC1 knockdown decreased ICAM-1 expression on mRNA level, whereas protein levels remained unchanged (Fig. 4I and K). Expression of the stress-responsive cell-cycle inhibitor p21 also remained unchanged following VKORC1 silencing (Fig. 4I).

Because VKORC1L1 represented the oxidant-responsive VKOR isoform in HCAEC, we next asked whether its expression adapts to VKORC1 loss. Indeed, VKORC1 loss triggered a 2.1-fold increase in VKORC1L1 expression (Fig. 4L). These data indicate that endothelial cells may compensate for reduced VKORC1 activity by up-regulating VKORC1L1. Together with the VKORC1L1 loss-of-function experiments, these findings support a model in which VKORC1L1 plays a central role in endothelial antioxidant defense and proliferative capacity, whereas VKORC1 appears less critical for endothelial redox homeostasis.

**A** qPCR validation of VKORC1 knock-down 24 h after siRNA transfection (*n* = 6). **B–D **AlamarBlue viability (*n* = 9), EdU proliferation (*n* = 6), and caspase-3/7 activity (*n* = 8) in HCAEC after VKORC1 silencing. **E **Scratch-wound migration over 12 h (*n* = 6). **F–G **Total ROS (DCFDA, *n* = 10, F) and RSL3-stimulated H₂O₂ release (Amplex Red, *n* = 6, G) following VKORC1 silencing. **H** Representative immunoblot of phosphorylated NF-κB and NF-κB after VKORC1 knockdown (*n* = 2) with β-actin loading control. **I** Representative immunoblot of p21 and ICAM-1 (*n* = 3) with β-actin loading control. **J**,** K**,** L** qPCR for NF-κB (*n* = 7), ICAM-1 (*n* = 10), and VKORC1L1 (*n* = 4) in VKORC1-deficient cells. Statistics: unpaired two-tailed t-test (A–D, F, G, J, K, L) or two-way ANOVA (E). Values are mean ± SEM; *P < 0.05, **P < 0.01.

## MK-7’s antioxidant and anti-inflammatory effects depend on VKORC1L1

Having shown that MK-7 promotes an anti-oxidative, pro-regenerative phenotype and that VKORC1L1 loss evokes the opposite response, we asked whether MK-7´s benefits require VKORC1L1 activity. HCAEC were first transfected with siRNAs against either VKORC1L1 or VKORC1 and then exposed to MK-7.

**Fig. 5:**
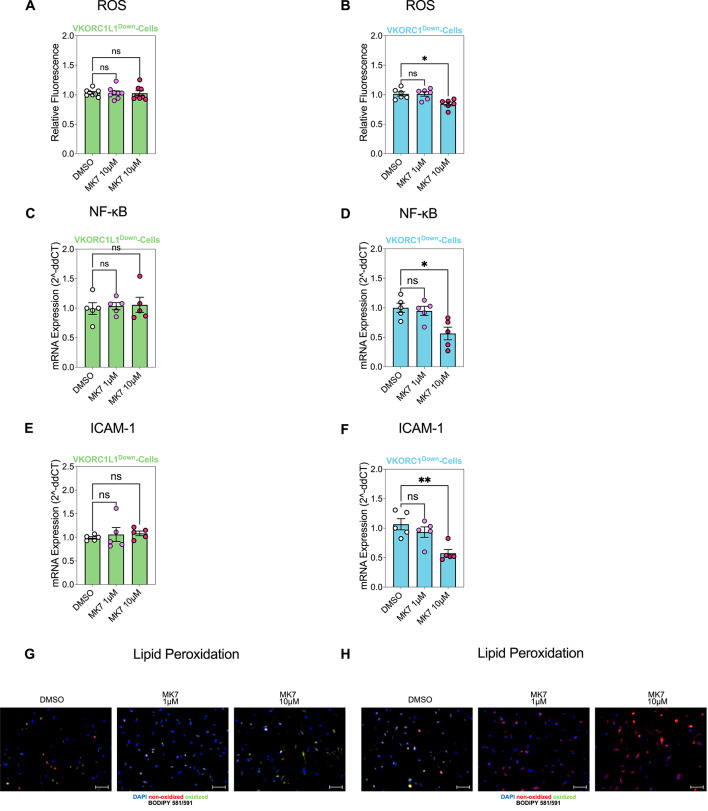
MK-7’s antioxidant and anti-inflammatory effects depend on VKORC1L1. **A–B **ROS production (DCFDA) after 24 h MK-7 treatment in HCAEC transfected with VKORC1L1 siRNA (A) or VKORC1 siRNA (B) (*n* = 6–8). **C–F **qPCR for NF-κB (C, D) and ICAM-1 (E, F) under the same conditions as A–B; VKORC1L1-silenced cells (left panels) versus VKORC1-silenced cells (right panels) (*n* = 5–7). **G–H **BODIPY-C11 fluorescence micrographs showing lipid peroxide accumulation after RSL3 (500 nM, 8 h) with or without MK-7 pre-treatment (24 h) in VKORC1L1-silenced (G) versus VKORC1-silenced (H) cells (*n* = 3). Statistics: one-way ANOVA + Dunnett’s multiple-comparison test. Data are mean ± SEM; *P < 0.05, **P < 0.01.

MK-7 did not have a detectable effect on ROS formation in VKORC1L1-depleted HCAEC (Fig. 5A), but efficiently lowered ROS levels in VKORC1-silenced cells (Fig. 5B). Measurements of NF-κB and ICAM-1 expression mirrored the ROS pattern: MK-7 failed to reduce NF-κB and ICAM-1 expression in VKORC1L1-deficient cells (Fig. 5C and E) but suppressed expression levels in VKORC1-knockdown cells (Fig. 5D and F). Finally, BODIPY 581/591 staining showed that MK-7 did not reduce RSL3-induced lipid peroxidation in VKORC1L1-silenced cells (Fig. 5G) whereas MK-7 reduced lipid peroxidation in VKORC1-silenced cells (Fig. 5H).

Together, these findings indicate that the antioxidant and anti-inflammatory effects of MK-7 in endothelial cells depend on VKORC1L1, but are largely independent of VKORC1. These data further support a central role for VKORC1L1 in vitamin K-mediated endothelial redox regulation.

**A–B **ROS production (DCFDA) after 24 h MK-7 treatment in HCAEC transfected with VKORC1L1 siRNA (A) or VKORC1 siRNA (B) (*n* = 6–8). **C–F **qPCR for NF-κB (C, D) and ICAM-1 (E, F) under the same conditions as A–B; VKORC1L1-silenced cells (left panels) versus VKORC1-silenced cells (right panels) (*n* = 5–7). **G–H **BODIPY-C11 fluorescence micrographs showing lipid peroxide accumulation after RSL3 (500 nM, 8 h) with or without MK-7 pre-treatment (24 h) in VKORC1L1-silenced (G) versus VKORC1-silenced (H) cells (*n* = 3). Statistics: one-way ANOVA + Dunnett’s multiple-comparison test. Data are mean ± SEM; *P < 0.05, **P < 0.01.

## MK-7 alleviates ER stress and subsequent endothelial inflammation in a VKORC1L1-dependent manner

Reactive oxygen species produced during oxidative protein folding render the endoplasmic reticulum a key redox hub in endothelial cells^31^. Given that VKORC1L1 resides in the ER membrane and promotes anti-oxidative capacity, we investigated whether VKORC1L1 connects vitamin K activity with ER-stress signaling. The ER stress inducer tunicamycin selectively upregulated VKORC1L1 (Figure S6A). Silencing VKORC1L1 modestly increased the key ER stress markers GRP78 and CHOP, indicating that loss of VKORC1L1 contributes to an early-stage ER stress response (Figure S6B – S6D). MK-7 treatment lowered tunicamycin-induced GRP78 and CHOP expression in a dose-dependent manner (Figure S6E – S6F) while also reducing tunicamycin-induced inflammatory activation (NF-κB/ICAM-1, Figure S6F – S6G). These protective effects on ER stress activation were abolished in VKORC1L1-silenced, but not VKORC1-silenced, cells (Figure S6I – S6J). Thus, VKORC1L1 deficiency is associated with ER stress, and VKORC1L1 activity appears to be required for MK-7 to limit ER stress and downstream inflammation.

### A Lipid peroxide/NF-κB/TNF axis drives inflammation in VKORC1L1-deficient HCAEC

**Fig. 6:**
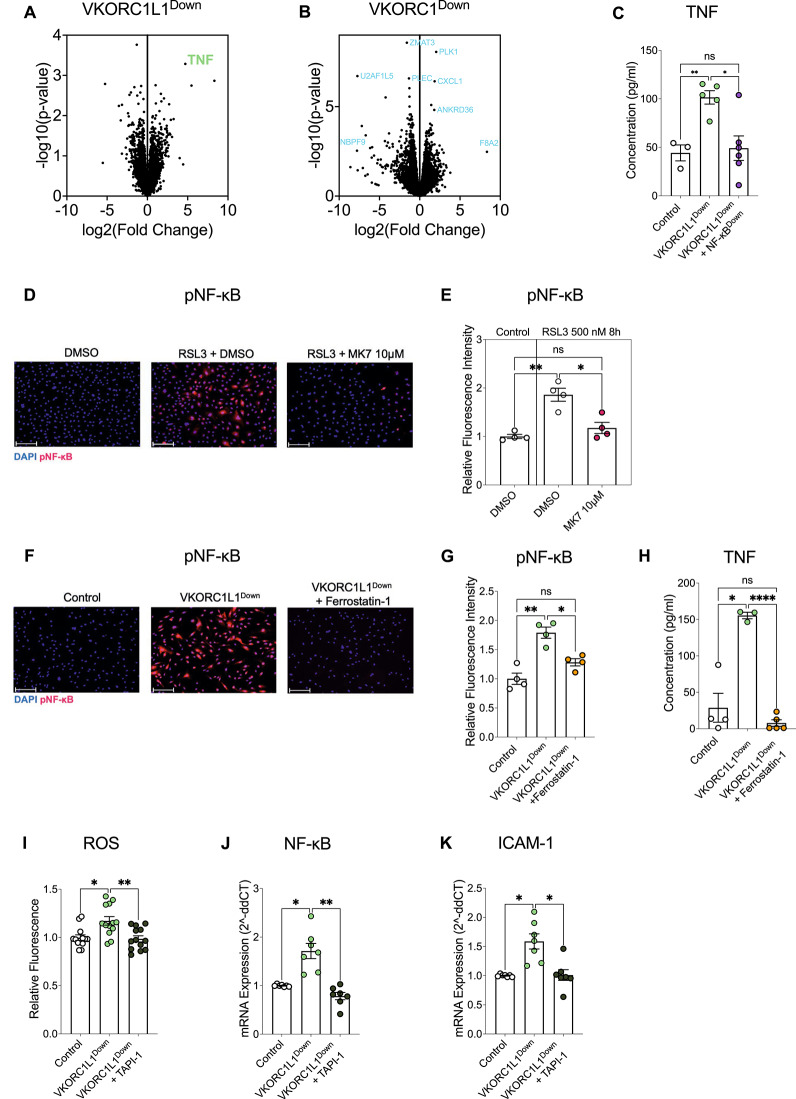
Secretion of tumor necrosis factor propagates inflammation in VKORC1L1-deficient HCAEC. **A–B **Bulk RNA-seq volcano plots after VKORC1L1 (A) or VKORC1 (B) knockdown in HCAEC. VKORC1L1 depletion markedly upregulates TNF. **C **Secreted TNF (ELISA) following VKORC1L1 silencing ± NF-κB co-silencing in LPS-stimulated cells (*n* = 3–6). **D–E **Representative phosphorylated NF-κB immunofluorescence and quantification upon RSL3-challenge (500nM, 8 h) ± MK-7 co-treatment (10µM) (*n* = 4=. **F–G** Representative phosphorylated NF-κB immunofluorescence after VKORC1L1 silencing ± ferrostatin-1 (1 µM, 24 h) in LPS-challenged cells (*n* = 4). **H **TNF release under the same treatments as G (*n* = 3–5). **I **ROS (DCFDA) after VKORC1L1 knockdown ± TAPI-1 (1 µM; TNF-shedding inhibitor) (*n* = 13). **J–K **qPCR for NF-κB and ICAM-1 in the experiment shown in I (*n* = 7 each). Statistics: one-way ANOVA with Dunnett’s multiple-comparison test for all panels. Data are mean ± SEM; *P < 0.05, **P < 0.01.

Bulk RNA-sequencing revealed that VKORC1L1 knockdown altered 186 transcripts, whereas VKORC1 knock-down affected 877 (Fig. 6A and B). KEGG pathway^32,33^ and gene ontology analysis of VKORC1-deficient cells highlighted oxidative phosphorylation and angiogenesis signatures, consistent with their pro-proliferative phenotype, whereas VKORC1L1-deficient cells lacked these shifts. Instead, VKORC1L1 silencing resulted in a marked 26-fold upregulation of TNF. Because TNF promotes endothelial ROS, ICAM-1 expression and cell death, we probed this finding further. The proximal TNF promoter is primarily regulated by NF-κB. In LPS-primed HCAEC, VKORC1L1 depletion markedly increased TNF secretion, an effect that was abolished when NF-κB was co-silenced (Fig. 6C).

Lipid peroxides, which accumulate after VKORC1L1 loss, have previously been described as pro-inflammatory^34^. Challenging HCAEC with the lipid peroxidation inducer RSL3 stimulated NF-κB activation, whereas MK-7 co-treatment attenuated this response (Fig. 6D and E). Likewise, the lipid-radical scavenger ferrostatin-1 suppressed both NF-κB activation and TNF release, supporting lipid peroxides as upstream mediators of TNF induction upon VKORC1L1 loss (Fig. 6F and H). Finally, inhibiting TNF shedding with TAPI-1 normalized ROS, NF-κB and ICAM-1 levels in VKORC1L1-silenced HCAEC, indicating that secreted TNF drives auto-/paracrine oxidative-inflammatory stress (Fig. 6I-K). Together, these findings support a model in which VKORC1L1 deficiency promotes a lipid-peroxide–NF-κB–TNF loop that amplifies endothelial inflammation, whereas vitamin K (via VKORC1L1) interrupts this cascade.

**A–B **Bulk RNA-seq volcano plots after VKORC1L1 (A) or VKORC1 (B) knockdown in HCAEC. VKORC1L1 depletion markedly upregulates TNF. **C **Secreted TNF (ELISA) following VKORC1L1 silencing ± NF-κB co-silencing in LPS-stimulated cells (*n* = 3–6). **D–E **Representative phosphorylated NF-κB immunofluorescence and quantification upon RSL3-challenge (500 nM, 8 h) ± MK-7 co-treatment (10 µM) (*n* = 4). **F–G** Representative phosphorylated NF-κB immunofluorescence after VKORC1L1 silencing ± ferrostatin-1 (1 µM, 24 h) in LPS-challenged cells (*n* = 4). **H **TNF release under the same treatments as G (*n* = 3–5). **I **ROS (DCFDA) after VKORC1L1 knockdown ± TAPI-1 (1 µM; TNF-shedding inhibitor) (*n* = 13). **J–K **qPCR for NF-κB and ICAM-1 in the experiment shown in I (*n* = 7 each). Statistics: one-way ANOVA with Dunnett’s multiple-comparison test for all panels. Data are mean ± SEM; *P < 0.05, **P < 0.01.

## Discussion

Our data support a vitamin K–VKORC1L1 axis that safeguards endothelial cells by limiting lipid peroxide accumulation, ferroptosis, and NF-κB/TNF–driven inflammation. MK-7 accelerated reendothelialization in mice and reduced ROS, lipid peroxidation, and cell death in vitro, effects that were lost when VKORC1L1 was silenced. These findings suggest that VKORC1L1 is an important mediator of vitamin K-dependent redox control and endothelial repair.

### VKORC1L1 is the redox-responsive vitamin K reductase in the endothelium

Previous reports showed that VKORC1L1 participates in antioxidant defense in smooth muscle cells and blocks ferroptosis in tumor lines, yet its endothelial role remained unknown^12–14^. We now demonstrate that oxidative stress selectively up-regulates VKORC1L1 (not VKORC1), and that VKORC1L1 loss alone is sufficient to trigger ER stress, ROS accumulation, growth arrest and inflammatory activation, phenotypic features that mirror early endothelial dysfunction in atherosclerosis. Conversely, VKORC1 knockdown lowered ROS and boosted proliferation, an effect possibly explained by a 2-fold compensatory increase in VKORC1L1 expression.

Several mechanisms may explain the divergent phenotypes observed after VKORC1L1 and VKORC1 silencing. First, the two enzymes likely serve distinct cellular functions. VKORC1 is best known for sustaining hepatic vitamin K recycling required for γ-carboxylation of coagulation factors, whereas VKORC1L1 appears to act as a stress-responsive redox enzyme in extra-hepatic cells. Second, VKORC1L1 was selectively induced by oxidative stress in our endothelial model, suggesting that it is expressed under conditions of increased redox demand. Finally, the increase in VKORC1L1 expression after VKORC1 knockdown suggests that endothelial cells may compensate for reduced VKORC1 activity by up-regulating VKORC1L1, thereby shifting vitamin K recycling toward antioxidant defense rather than classical γ-carboxylation. Together, these data indicate that VKORC1L1 represents the redox-responsive arm of the vitamin K cycle in endothelial cells, whereas VKORC1 appears less central for endothelial antioxidant protection. However, the precise molecular determinants that confer these distinct functions to VKORC1L1 and VKORC1 remain incompletely defined and will require further investigation.

### A lipid-peroxide–NF-κB–TNF loop drives injury when VKORC1L1 is absent

RNA-sequencing highlighted TNF as a highly up-regulated transcript (26-fold) after VKORC1L1 silencing. We show that lipid peroxides accumulating under VKORC1L1 deficiency activate NF-κB, which in turn drives TNF transcription. Secreted TNF then reinforces ROS production, ICAM-1 expression and further NF-κB activation, creating a self-amplifying inflammatory loop. Increased oxidative stress within this inflammatory circuit is likely a major contributor to the impaired endothelial proliferation and cellular stress responses observed after VKORC1L1 silencing. Three intervention points corroborate this mechanism (i) MK-7 or ferrostatin-1 (lipid-radical scavenger) blocked NF-κB activation and TNF release. (ii) NF-κB knockdown prevented the rise in TNF. (iii) Inhibiting TNF shedding with TAPI-1 normalized ROS, NF-κB and ICAM-1 in VKORC1L1-deficient cells. Consistent with our findings, previous studies in murine lung injury models and microglia have demonstrated that vitamin K2 inhibits TNF expression upon pro-inflammatory stimulation with LPS^35,36^. Taken together, our data support a model in which VKORC1L1 restrains a lipid-peroxide/NF-κB/TNF axis that otherwise propagates oxidative and inflammatory stress.

### Vitamin K and cellular antioxidation

While vitamin K’s canonical role in coagulation is well established, more recent work revealed its antioxidant and anti-calcific properties^6,37–40^. Bar et al.^19^ showed that MK-7 augments endothelial NO production and vasodilation, while Mishima et al.^21^ identified vitamin K as a ferroptosis suppressor. Our data extend these observations by showing that: (i) MK-7 promotes endothelial regeneration through lipid peroxide control; (ii) VKORC1L1, not VKORC1, provides the enzymatic reduction required for this task in the endothelium; and (iii) inhibition of ER stress and TNF release are additional layers of MK-7-mediated protection. These data align with VKORC1L1´s intracellular localization at the ER, a central site of lipid peroxidation^41,42^. Disruption of ER-associated redox homeostasis may thereby contribute to ER stress activation, impaired proliferation, and inflammatory stress responses in endothelial cells lacking VKORC1L1.

### Translational implications

Population cohorts link higher vitamin K intake to lower incidence of coronary events, valve calcification and cardiac mortality^43–45^. MK-7 is orally bioavailable, safe even at pharmacologic doses and, critically, does not modulate coagulation at concentrations that protect endothelium in our study^5,46–48^. The present work provides mechanistic support for these epidemiologic findings and suggests that MK-7 or future VKORC1L1-targeted activators could complement existing strategies aimed at limiting oxidative and inflammatory vascular injury.

### Limitations and future directions

We employed acute carotid denudation as an in-vivo read-out. Confirming VKORC1L1 dependence in a chronic atherosclerosis or hypertension model will be important and CRISPR-based endothelial-specific VKORC1L1 knock-out mice would provide genetic proof in vivo. Moreover, clinical studies should examine whether systemic and vascular vitamin K status correlate with endothelial VKORC1L1 activity and cardiovascular outcomes. In addition, although our data support a central role of VKORC1L1 in limiting ROS, lipid peroxidation and inflammatory signaling, the precise molecular mechanisms by which VKORC1L1 regulates local redox balance remain to be fully elucidated.

In summary, vitamin K, via VKORC1L1, quenches lipid peroxides, prevents ferroptosis, and interrupts an NF-κB/TNF inflammatory circuit, thereby promoting endothelial repair. These insights characterize a novel antioxidant and anti-inflammatory branch of the vitamin K cycle in the endothelium and highlight MK-7 as a promising, low-risk therapeutic candidate for diseases driven by endothelial dysfunction.

.

## Materials and methods

### Cell Culture and Treatments

Primary human coronary artery endothelial cells (HCAEC; PromoCell C-12221), of both sexes (passages 6–8), were maintained in endothelial growth medium (PromoCell C-22020; 5% FBS) at 37 °C in 5% CO₂. For vitamin K supplementation, cells were treated with MK-7 (1–10 µM; Santa Cruz sc-218691) or phylloquinone (K1, Sigma V3501) (dissolved in DMSO) for 24 h. Where indicated, HCAEC were co-incubated with EndMT differentiation medium (base medium + 10 % FBS, 1 µg/ml hydrocortisone and ascorbic acid, 10 ng/ml TGF-β₂ and IL-1β; PeproTech) for 96 h (refreshed every 48 h), tunicamycin (5 µg/ml; Sigma T7765), oxidized LDL (50 µg/ml; Thermo L34357), LPS (1 µg/ml for 4 h; Sigma L2630), or ferrostatin-1 (1 µM for 24 h; MedChemExpress HY-100579). For gene silencing, HCAEC were transfected in Opti-MEM with Lipofectamine RNAiMAX and 10 nM siRNA targeting VKORC1L1 (Thermo 4392420), VKORC1 (AM16708), or a non-targeting control (AM4611). Assays were performed 24 h post-transfection.

### Viability, proliferation, apoptosis, and migration assay

Cell viability was measured by adding AlamarBlue™ HS (Thermo A50100) for 4 h (protected from light) and reading absorbance at 570 nm and 600 nm (Infinite M200, Tecan). Proliferation was assessed with the Click-iT™ EdU Microplate Assay (Thermo C10499): cells were pulsed with 10 µM EdU for 2 h before fixation. They were then incubated with HRP-conjugated click reagent, followed by Amplex UltraRed. The resulting fluorescence was quantified (Ex 568 nm/Em 585 nm). Apoptosis was determined by the Caspase-Glo^®^ 3/7 assay (Promega G8091): the reagent was added for 2 h at room temperature before recording luminescence produced by luciferase. The resulting signal is proportional to the amount of caspase 3/7 activity. For migration, confluent cells in 12-well plates were scratched with a 100 µl pipette tip, washed, then incubated in 2.5% serum medium to minimize proliferation. Images were captured at 0, 4, 8, and 12 h (Axio Observer 7) and wound closure was quantified.

### Reactive oxygen species (ROS) and lipid peroxidation

Total ROS were measured by incubating cells with 50 µM DCFDA (Sigma D6883) for 45 min, washing with PBS, and immediately reading fluorescence (Ex 492 nm/Em 527 nm). H₂O₂ release was quantified using the Amplex Red kit (Thermo A22188): 50 µl conditioned medium plus 50 µl HRP (0.1 U/ml) and Amplex Red (50 µM) mixture incubated 1 h at 37 °C in the dark before measurement of fluorescence (Ex 560 nm/Em 590 nm). Lipid peroxidation was induced by RSL3 (500 nM, 8 h; MedChemExpress 1219810-16-8) and detected by BODIPY 581/591 C11 (Thermo Fisher Scientific, Cat# D3861) staining. Cells in 6-well plates were labeled with 2 µM BODIPY for 30 min, trypsinized, strained, resuspended in FACS buffer, and analyzed by flow cytometry (FITC/Texas Red channels on a BD LSRFortessa^TM^ cell analyzer). In another approach, cells seeded on coverslips were BODIPY labeled, fixed, and then mounted with DAPI medium (Vectashield, Vector Laboratories, H-1200-10) before imaging via fluorescence microscopy (Axio Observer 7). The fluorescence intensity of oxidized lipids was analyzed through the FITC channel and the intensity of reduced lipids through the Texas Red channel.

### Quantitative real-time polymerase chain reaction

Total RNA was extracted with TRIzol™ (Thermo 15596018), quantified by NanoDrop, and 0.5–2 µg was reverse-transcribed (Qiagen Omniscript). qPCR was performed on a QuantStudio 3 or 7500 HT (Applied Biosystems) using TaqMan assays (Thermo) and Gene Expression Master Mix with 18 S as the reference gene. All reactions were run in triplicate, and data analyzed by the ΔΔCT method. The following TaqMan Assays were used: VKORC1L1 (Hs04989728_s1), VKORC1 (Hs00829655_s1), nuclear factor ‘kappa-light-chain-enhancer’ of activated B-cells (NF-κB, Hs00765730_m1), glucose-regulated protein 78 kDa (GRP78, Hs00607129_gH), C/EBP homologous protein (CHOP, Hs00358796_g1) and 18 S ribosomal RNA (18 S, Hs99999901_s1), ICAM-1 (Hs00164932_m1), VCAM-1 (Hs00365486_m1), PECAM1 (Hs01065279_m1), eNOS (NOS3, Hs01574665_m1), CDH5 (Hs00901465_m1), N-Cadherin (CDH2, Hs00983056_m1), SM22 (Transgelin, Hs00162558_m1), Calponin 1 (Hs00959434_m1).

#### Protein analysis

For immunoblotting, cells were lysed in RIPA with protease/phosphatase inhibitors (Roche, Cat# 4693132001, and Roche, Cat# 4906845001). Protein concentration was measured by Qubit™. 50 µg protein per lane were separated on SDS-PAGE (Bio-Rad precast gels, Cat#456–1084). Proteins were transferred to nitrocellulose membranes (Carl Roth GmbH, HP40.1), blocked in 5% BSA, and probed overnight at 4 °C with primary antibodies, followed by HRP-conjugated secondary antibodies and chemiluminescent detection (Sigma GERPN2232) on a ChemiDoc system. Following antibodies were used: mouse anti-β-Actin antibody (Sigma-Aldrich, Cat# A1978, 1:2000), rabbit p-NF-κB (Cell Signaling Technology, Cat# 3033 S, 1:1000), rabbit NF-κB (Cell Signaling Technology, Cat# 8242 S, 1:1000), rat anti-mouse-IgG antibody (Sigma-Aldrich, Cat# A9044, 1:2000), and HRP-conjugated goat anti-rabbit-IgG (Cell Signaling Technology, Cat# 7074 S, 1:1000).

Immunofluorescence was carried out on PFA-fixed (4% PFA, 30 min, room temperature), and then Triton-permeabilized (0.25% Triton X-100, 10 min, room temperature) cells. Cells were then blocked in 1% BSA-glycine in PBS-T (0.1% Tween20) for 30 min at room temperature. Primary antibodies were applied overnight, followed by Cy2/Cy3 secondary antibodies, and mounting with DAPI medium. Imaging was performed on an Axio Observer 7. The following primary antibodies were used: VKORC1L1 (Sigma-Aldrich, Cat# HPA053954, 1:100), VKORC1 (Sigma-Aldrich, Cat# HPA042720, 1:100), TAGLN (Abcam, Cat#14106, 1:200), VWF (Abcam, Cat#6994, 1:200), CD31 (Abcam, Cat #28364, 1:100), Vimentin (Abcam, Cat#8978, 1:700), eNOS (Abcam, Cat#ab76198, 1:100), ICAM-1 (Santa Cruz, #sc-8439, 1:100), VCAM-1 (Abcam Cat#134047, 1:100), p-NF-κB (Cell Signaling Technology, Cat# 3033 S, 1:100), N-Cadherin (Cell Signaling Technology, Cat# 13116 T, 1:100), VE-Cadherin (Cell Signaling Technology, Cat# 2500 T, 1:100), Goat Anti-rabbit IgG Cy2 (Dianova, 1:100), and Goat Anti-mouse IgG Cy3 (Dianova, 115–165-146, 1:100).

For the analysis of extracellular proteins, the cell medium was collected and centrifuged at 800 × g for 8 min. The resulting supernatant was analyzed by using commercially available enzyme-linked immunosorbent assay (ELISA) kits (Human IL-6 ELISA MAX™ Deluxe 5 Plates, BioLegend, Cat# 430504 or BD OptEIA™ Human TNF ELISA Kit II, BD Biosciences, Cat# 550610). Four-parameter logistic regression (GraphPad Prism 10) was used to plot the concentrations.

### Next-generation sequencing

3’ mRNA Sequencing was performed at the next generation sequencing core facility of the medical faculty Bonn using the Lexogen QuantSeq 3’mRNA FWD Library Prep Kit with 200 ng total RNA as input material. Sequencing was performed on an Illumina NovaSeq 6000 device at 10 million reads per sample. Sequencing data are available in ArrayExpress.

#### Animal experiments

Carotid artery injury was performed as previously described^49^. Briefly, male and female C57BL/6J mice (Janvier) were fed a diet enriched with MK-7 (100 µg MK-7/g diet, adapted from Scheiber et al.^50^) or a regular chow diet starting two days before surgery (*n* = 8 mice per group; 4 females and 4 males). Both diets included 7 µg/g diet of menadione nicotinamide bisulfite (vitamin K3), a commonly used vitamin K source in rodent diets. Animals were randomly assigned to dietary groups with balanced allocation by sex. All surgical procedures, outcome assessments, and data analyses were performed blinded to dietary assignment. MK-7 was a generous gift by Gnosis by Lesaffre (Marcq-en-Baroeul, France) and incorporated into standard chow by ssniff-Spezialdiäten GmbH (Soest, Germany). Mice were anesthetized with intraperitoneal injections of 100 mg/kg ketamine and 16 mg/kg xylazine. A small incision was made on the left neck, and the left common carotid artery was carefully exposed up to the bifurcation. Endothelial denudation was performed by applying three consecutive 5-second bursts of 2 W through 2-mm-wide forceps. The incision was then sutured, and the mice were allowed to recover. On day 5, the mice were re-anesthetized using the same ketamine-xylazine protocol, 200 µl of blood were collected from the inferior vena cava and 50 µL of Evans blue solution was injected intravenously into the inferior vena cava. Following a two-minute circulation period, transcardiac perfusion with 0.9% NaCl was performed to remove unbound dye. The mice were euthanized by caval bleeding under anesthesia, and both common carotid arteries were excised. Residual connective tissue was carefully removed, and images were taken using a Zeiss Zen Observer 7 microscope. The total lesion area and the remaining denuded area (stained blue) were quantified with Zen software. Reendothelialization was expressed as 1 minus the fraction of the remaining denuded area, multiplied by 100 to yield a percentage.

### Statistical analysis

Methods and details for statistics of individual experiments are presented in the corresponding figure legends. All data points in the presented figures represent biological replicates. Means of two groups were compared with an unpaired t-test after testing for normal distribution. Means of more than two groups were compared by an unpaired one-way ANOVA followed by Dunnett´s multiple comparison test or by two-way ANOVA. All reported p-values are two-sided. Statistical analyses were performed using the software GraphPad Prism 10.

#### Study approval

Our study received the proper ethical oversight. All animal experiments complied with all institutional and national requirements for the care and use of laboratory animals (German Animal Protection Law) and received committee approval from the National Office for Nature, Environment and Consumer Protection in Recklinghausen, North Rhine-Westphalia (permit number: 81-02.04.04.2023.A390).

## Supplementary Information

Below is the link to the electronic supplementary material.

Supplementary Material 1

Supplementary Material 2

Supplementary Material 3

Supplementary Material 4

## Data Availability

The datasets generated during the current study are available in the ArrayExpress repository (Accession number: E-MTAB-16661,(https:/www.ebi.ac.uk/biostudies/arrayexpress/studies/E-MTAB-16661)).Other data are available upon reasonable request.

## Abbreviations

EndMT: Endothelial-to-mesenchymal-transition
ER: Endoplasmic reticulum
HCAEC: Human coronary artery endothelial cells
MK-7: Menaquinone-7
NF-κB: Nuclear factor 'kappa-light-chain-enhancer’ of activated B cells
ROS: Reactive oxygen species
TNF: Tumor necrosis factor
TGF-β: Transforming growth factor beta
VKORC1: Vitamin-K-2,3-epoxide-reductase-complex-subunit-1
VKORC1L1: Vitamin-K-2,3-epoxide-reductase-complex-subunit-1-like-1

## Declarations

### Total words

4489 (excluding the abstract, figure legends and references).
