# Supplementary material for: VKORC1L1–mediated vitamin K recycling counters ferroptosis to promote endothelial repair
Source: Sci Rep. 2026 Jun 19;16:19171. doi: 10.1038/s41598-026-54463-7 (PMC13282403; doi:10.1038/s41598-026-54463-7)
Supplement: Supplementary file 1 — Supplementary Material 1 [file 41598_2026_54463_MOESM1_ESM.pdf]

Figure Legends

Figure 1 Vitamin K1 protects against ferroptosis

A–B Cell viability (AlamarBlue) and apoptosis (caspase-3/7) in HCAEC after 24 h K1 (1–10 µM); n = 8 (A) and 7 (B). **C** Viability after ferroptosis induction with RSL3 (500 nM, 8 h) ± K1; n = 8. Statistics: one-way ANOVA + Dunnett (A-C); Data are mean ± SEM. *P < 0.05; **P < 0.01; ***P < 0.001; ****P < 0.0001.

Figure 2 Vitamin K2 (MK-7) limits endothelial-to-mesenchymal transition (EndMT)

A Bright-field morphology of HCAEC cultured 96 h in EndMT medium ± MK-7 (5 µM); scale 100 µm; n = 3. **B–E** Immunofluorescence (left) and per-cell quantification of mesenchymal markers after 96 h EndMT ± MK-7: SM22 (C), Vimentin (D), N-cadherin (E); scale 200 µm; n = 3–4. **F–J** Endothelial markers under the same conditions: eNOS (F), CD31 (G), vWF (H), VE-cadherin (I); scale 200 µm; n = 3. **K–L** qPCR for VKORC1L1 (I) and VKORC1 (J) following 96 h EndMT; n = 6 (K) and 5 (L). **M** VKORC1L1 (left) and VKORC1 (right) immunofluorescence after EndMT; scale 200 µm; n = 3. Statistics: one-way ANOVA + Dunnett for B–J; unpaired two-tailed t-test for J–K Data are mean ± SEM; *P < 0.05, **P < 0.01, ***P < 0.001, ****P < 0.0001.

Figure 3 Vitamin K1 mirrors MK-7 in inhibiting EndMT

A Immunofluorescence of mesenchymal markers after 96 h EndMT ± K1: SM22, Vimentin, N-cadherin. scale 200 µm; n = 3–4. **B** Immunofluorescence of endothelial markers after 96 h EndMT ± K1: eNOS, CD31, vWF, VE-Cadherin. scale 200 µm; n = 3–4.

Figure 4 VKOR-enzyme knockdowns

A–B qPCR validation of VKORC1L1 and VKORC1 knock-down 24 h post-siRNA; n = 6. **C** IL-6 release (ELISA) 24 h after VKORC1L1 knock-down; n = 5. Statistics: unpaired t-test (all panels); mean ± SEM; *P < 0.05, **P < 0.01.

Figure 5 ER stress links VKORC1L1 to endothelial inflammation

A Dose-dependent induction of VKORC1L1 (but not VKORC1) mRNA by tunicamycin (0.1-5 μg ml^{-1}, 6 h); n = 5. **B-C** qPCR for GRP78 and CHOP up- 24 h after VKORC1L1 silencing; n = 5. **D-G** MK-7 pre-treatment (0.1–10 μM, 24 h) attenuates tunicamycin-induced GRP78, CHOP, NF-κB and ICAM-1; n = 5-7. **H-I** MK-7 fails to suppress GRP78 when VKORC1L1 is depleted; n = 4. Statistics: unpaired two-tailed t-test (A–C, E–K) or two-way ANOVA (D). Values are mean ± SEM; *P < 0.05, **P < 0.01.

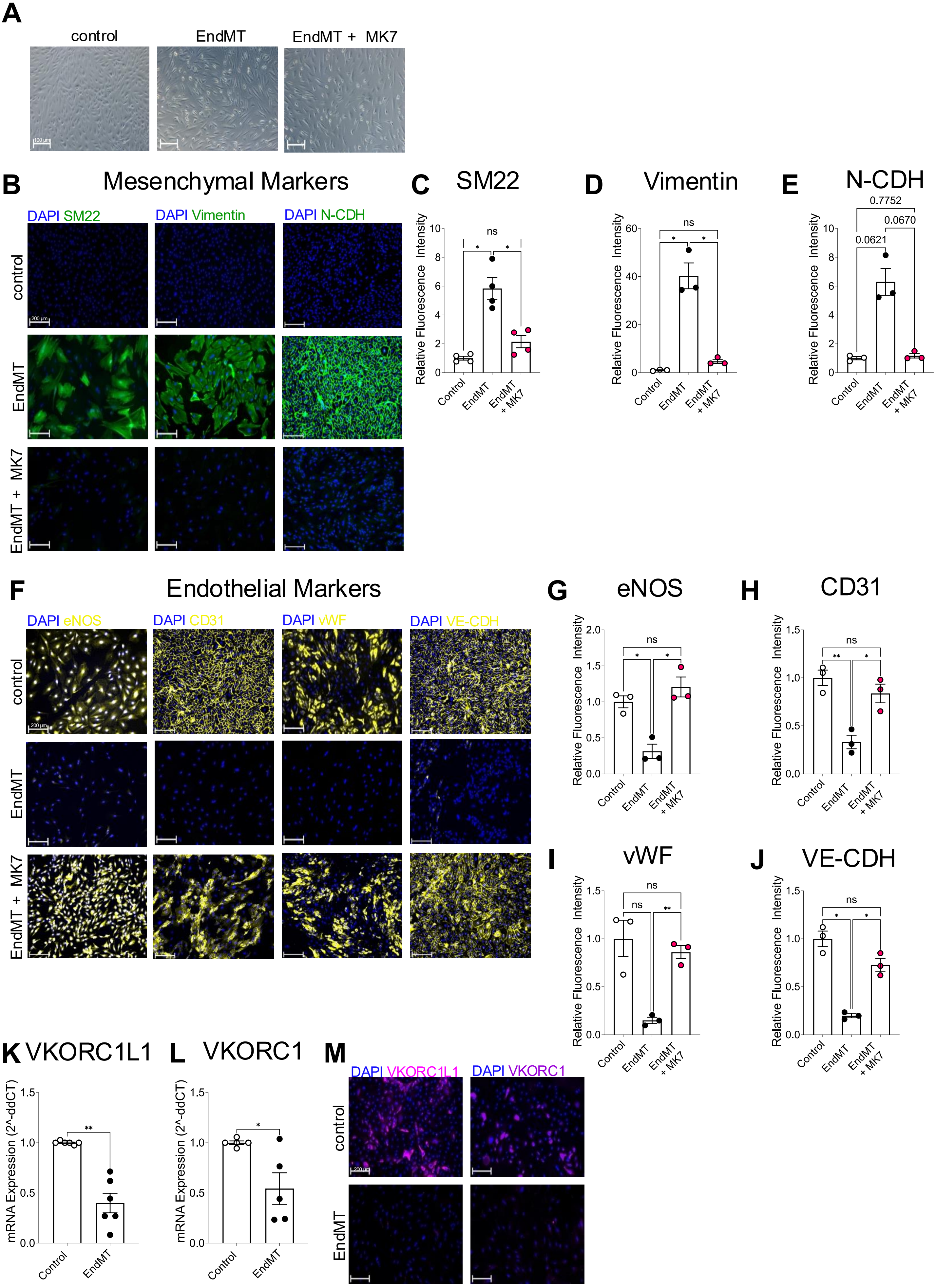

A
control
EndMT
EndMT + MK7
B
Mesenchymal Markers
DAPI SM22
DAPI Vimentin
DAPI N-CDH
control
EndMT
EndMT + MK7
C
SM22
D
Vimentin
E
N-CDH
Relative Fluorescence Intensity
ns
0.7752
0.0670
0.0621
Control
EndMT
EndMT + MK7
F
Endothelial Markers
DAPI eNOS
DAPI CD31
DAPI vWF
DAPI VE-CDH
G
eNOS
H
CD31
I
vWF
J
VE-CDH
K
VKORC1L1
L
VKORC1
mRNA Expression (2^-ddCT)
M
DAPI VKORC1L1
DAPI VKORC1

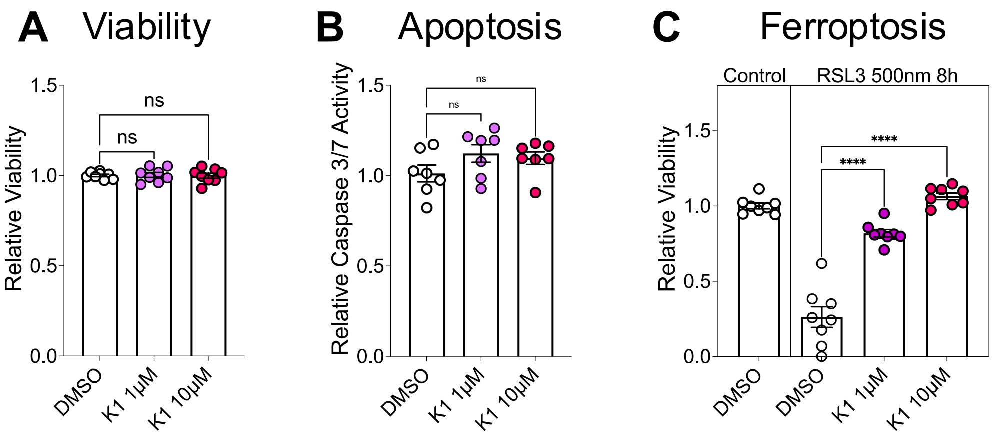
A
Viability
B
Apoptosis
C
Ferroptosis
Relative Viability
1.5
1.0
0.5
0.0
ns
ns
DMSO
K1 1µM
K1 10µM
Relative Caspase 3/7 Activity
ns
ns
Control
RSL3 500nm 8h
****
****

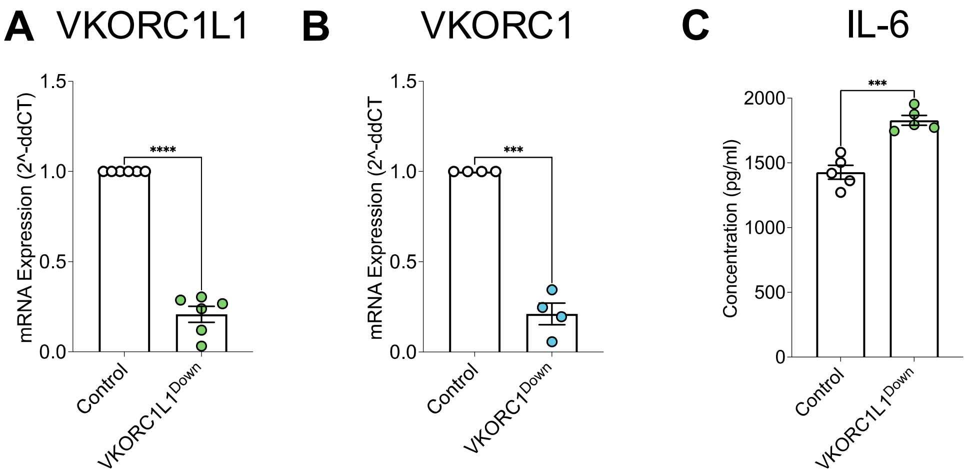

A
VKORC1L1
mRNA Expression (2^-ddCT)
1.5
1.0
0.5
0.0
****
Control
VKORC1L1Down
B
VKORC1
mRNA Expression (2^-ddCT
1.5
1.0
0.5
0.0
***
Control
VKORC1Down
C
IL-6
Concentration (pg/ml)
2000
1500
1000
500
0
***
Control
VKORC1L1Down

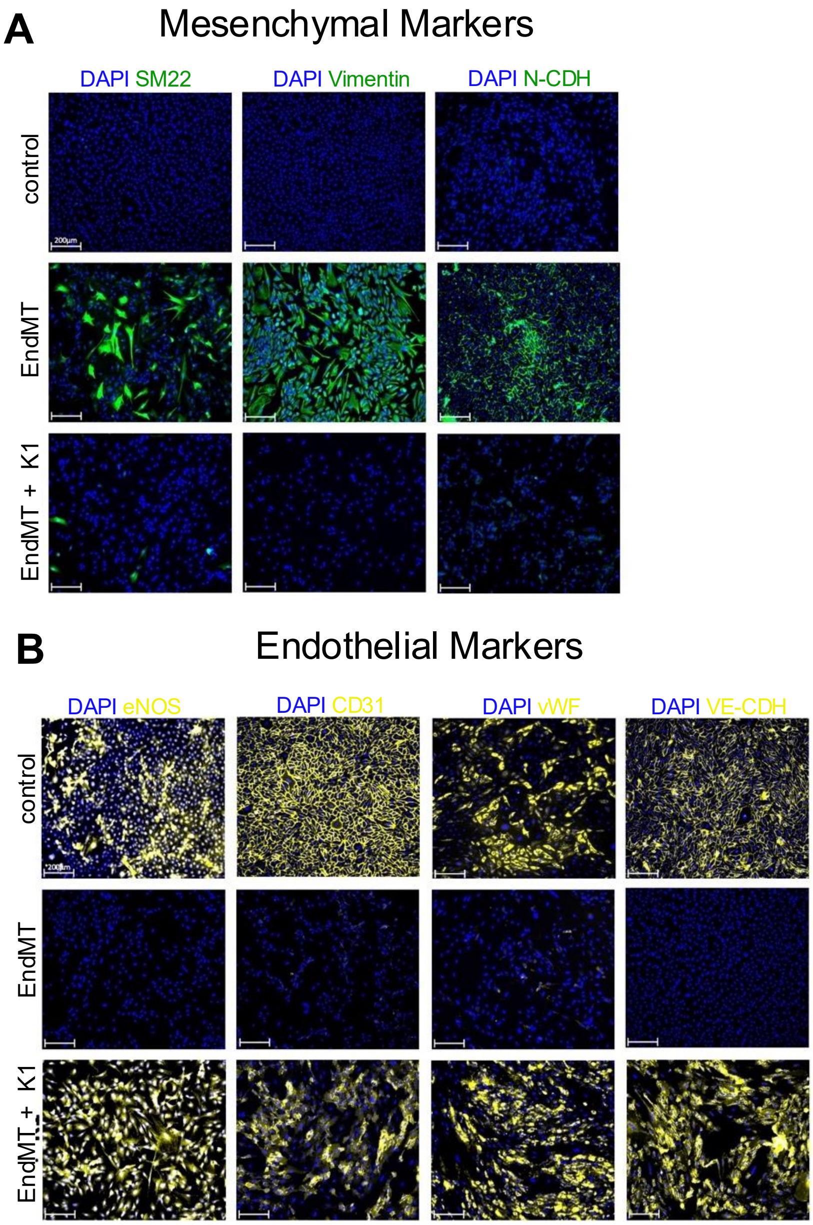

A
Mesenchymal Markers
DAPI SM22
DAPI Vimentin
DAPI N-CDH
control
EndMT
EndMT + K1
200μm
B
Endothelial Markers
DAPI eNOS
DAPI CD31
DAPI vWF
DAPI VE-CDH
control
EndMT
EndMT + K1
200μm

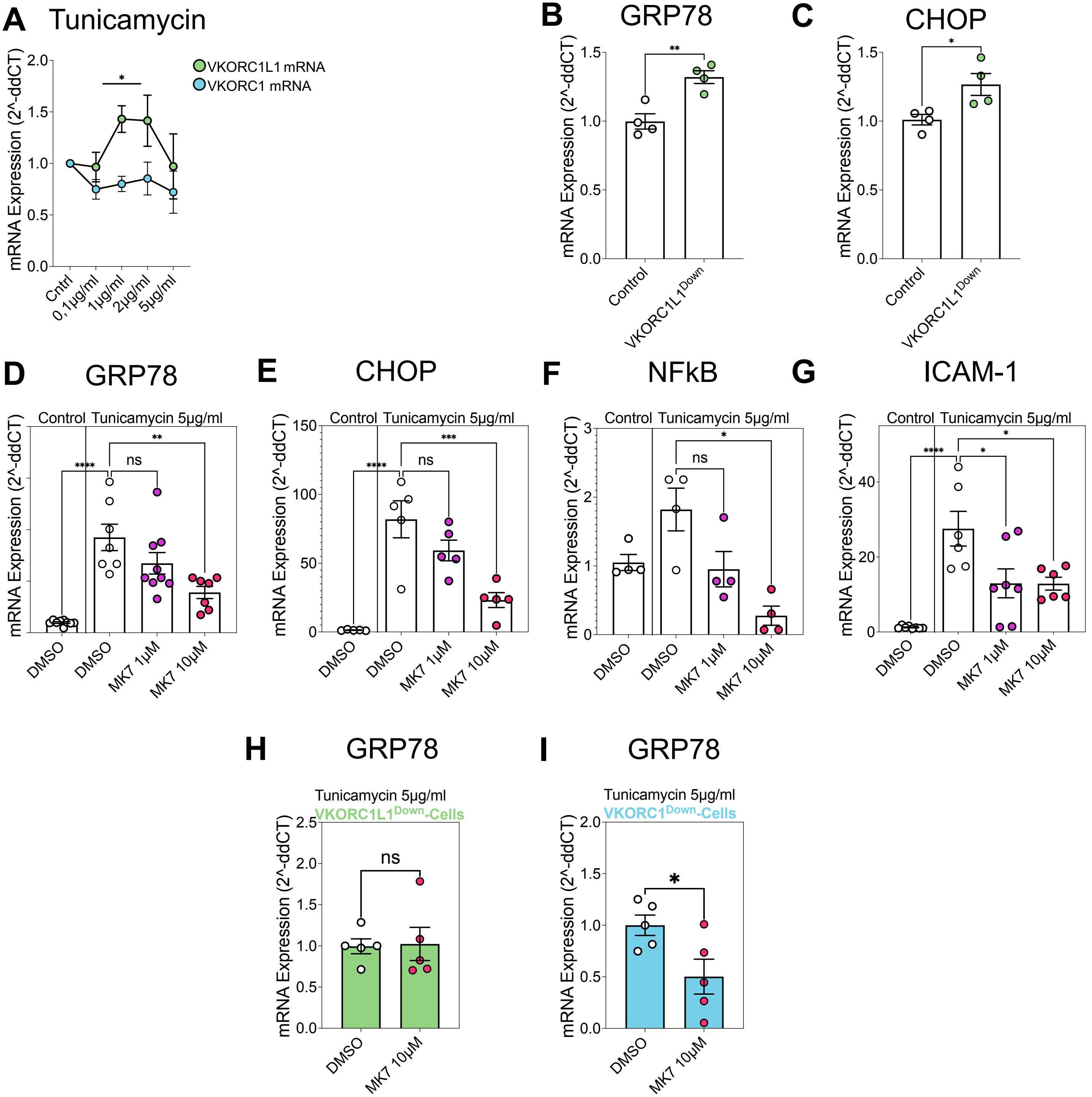
A
Tunicamycin
VKORC1L1 mRNA
VKORC1 mRNA
mRNA Expression (2^-ddCT)
2.0
1.5
1.0
0.5
0.0
*
Cntrl
0,1µg/ml
1µg/ml
2µg/ml
5µg/ml
B
GRP78
**
mRNA Expression (2^-ddCT)
1.5
1.0
0.5
0.0
Control
VKORC1L1 Down
C
CHOP
*
mRNA Expression (2^-ddCT)
1.5
1.0
0.5
0.0
Control
VKORC1L1 Down
D
GRP78
Control Tunicamycin 5µg/ml
****
ns
**
mRNA Expression (2^-ddCT)
DMSO
DMSO
MK7 1µM
MK7 10µM
E
CHOP
Control Tunicamycin 5µg/ml
****
ns
***
mRNA Expression (2^-ddCT)
150
100
50
0
DMSO
DMSO
MK7 1µM
MK7 10µM
F
NFkB
Control Tunicamycin 5µg/ml
ns
*
mRNA Expression (2^-ddCT)
3
2
1
0
DMSO
DMSO
MK7 1µM
MK7 10µM
G
ICAM-1
Control Tunicamycin 5µg/ml
****
*
*
mRNA Expression (2^-ddCT)
40
20
0
DMSO
DMSO
MK7 1µM
MK7 10µM
H
GRP78
Tunicamycin 5µg/ml
VKORC1L1 Down -Cells
ns
mRNA Expression (2^-ddCT)
2.5
2.0
1.5
1.0
0.5
0.0
DMSO
MK7 10µM
I
GRP78
Tunicamycin 5µg/ml
VKORC1 Down -Cells
*
mRNA Expression (2^-ddCT)
2.0
1.5
1.0
0.5
0.0
DMSO
MK7 10µM
