# Supplementary material for: VKORC1L1–mediated vitamin K recycling counters ferroptosis to promote endothelial repair
Source: Sci Rep. 2026 Jun 19;16:19171. doi: 10.1038/s41598-026-54463-7 (PMC13282403; doi:10.1038/s41598-026-54463-7)

Uncropped Blots

Figure 1H

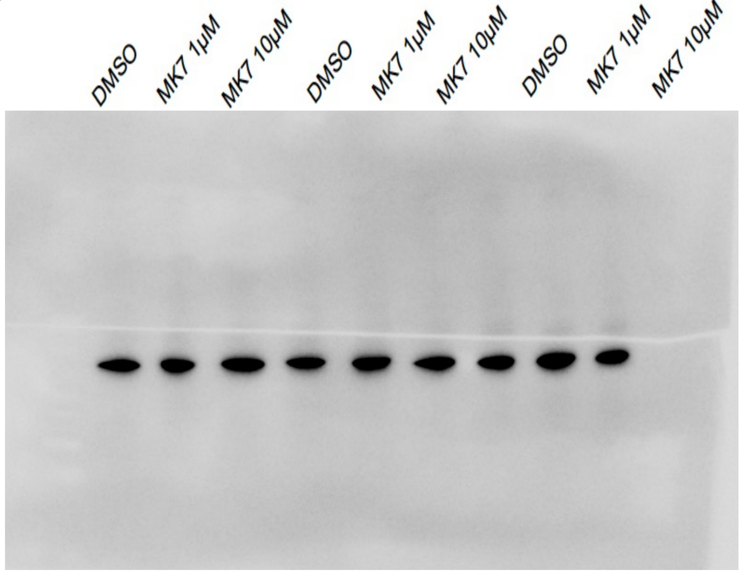

β-actin

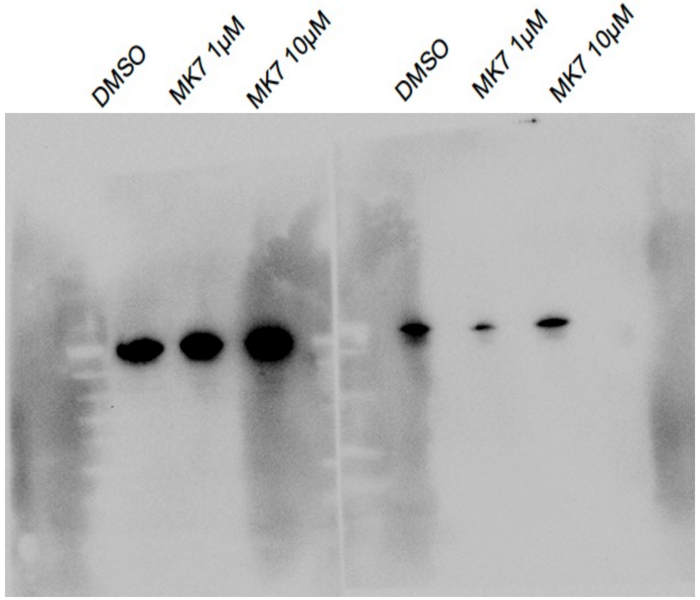

NF-κB pNF-κB

Uncropped Blots

Figure 1H – Other replicates (not shown in Paper)

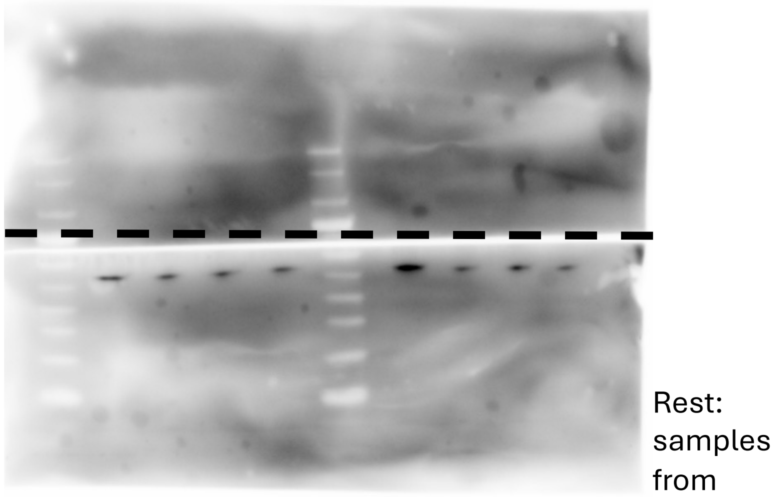

β-actin

Rest: samples from unrelated other project

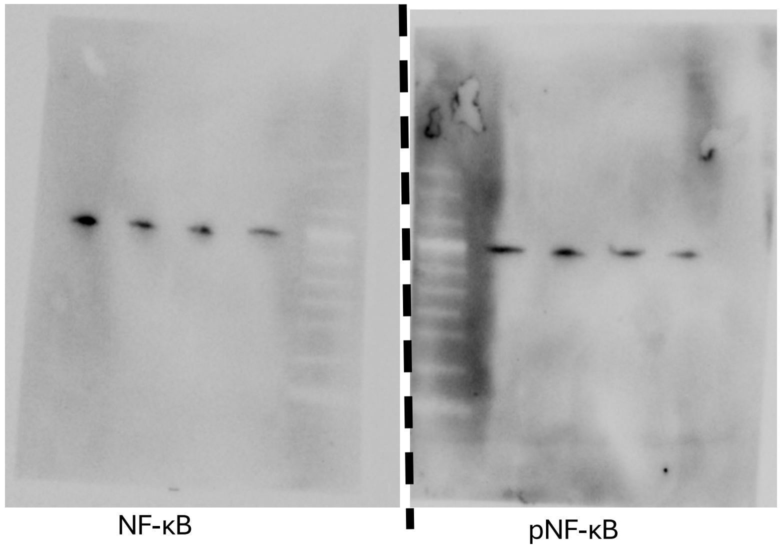

NF-κB

pNF-κB

Uncropped Blots

Figure 3J & 4H

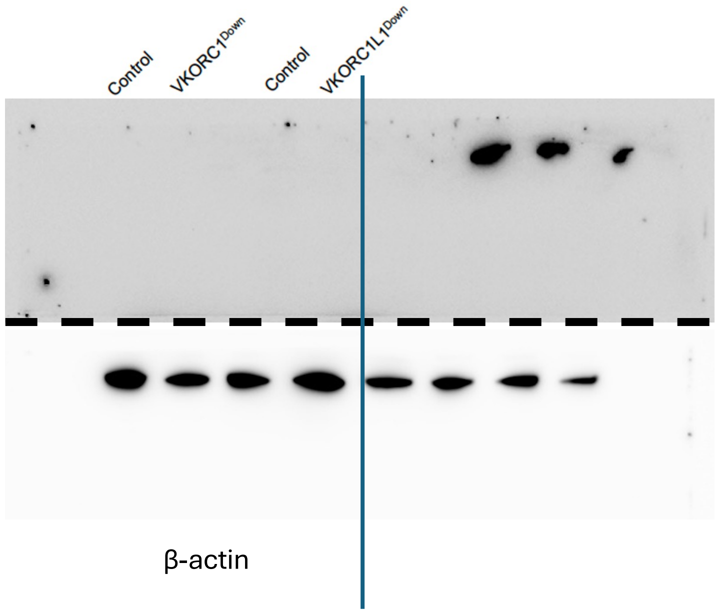

Rest: samples from unrelated other project

β-actin

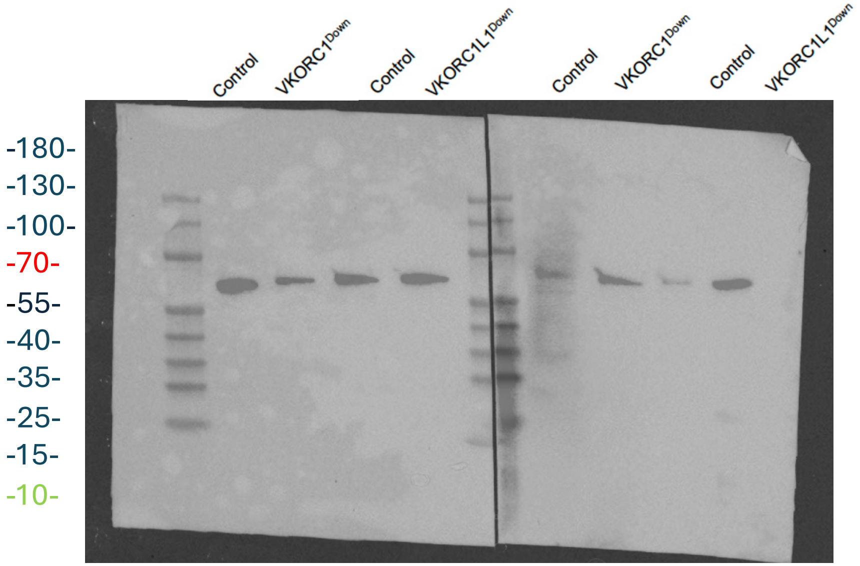

NF-κB

pNF-κB

Uncropped Blots

Figure 3J & 4H

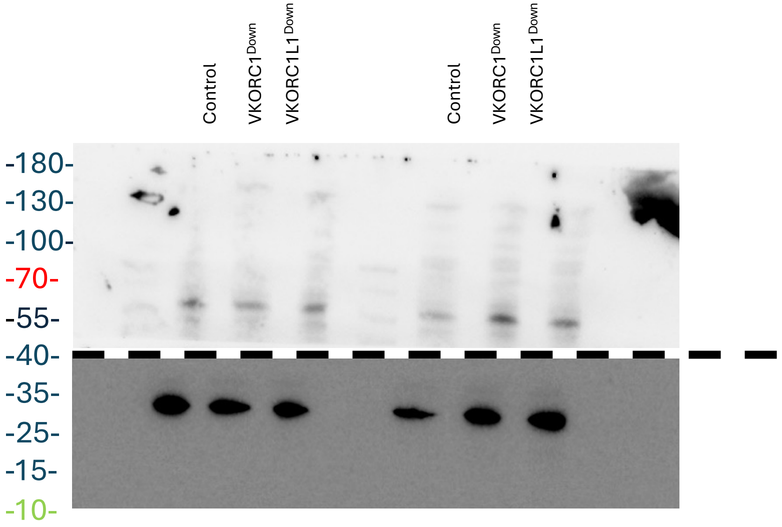

β-actin

Rest: proteins for unrelated other project

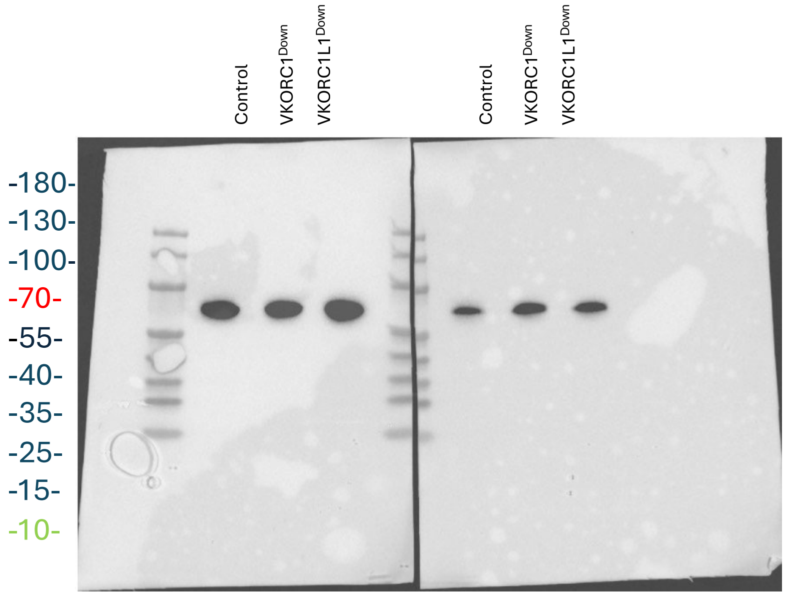

NF-κB

pNF-κB

Uncropped Blots

Figure 3K & 4I

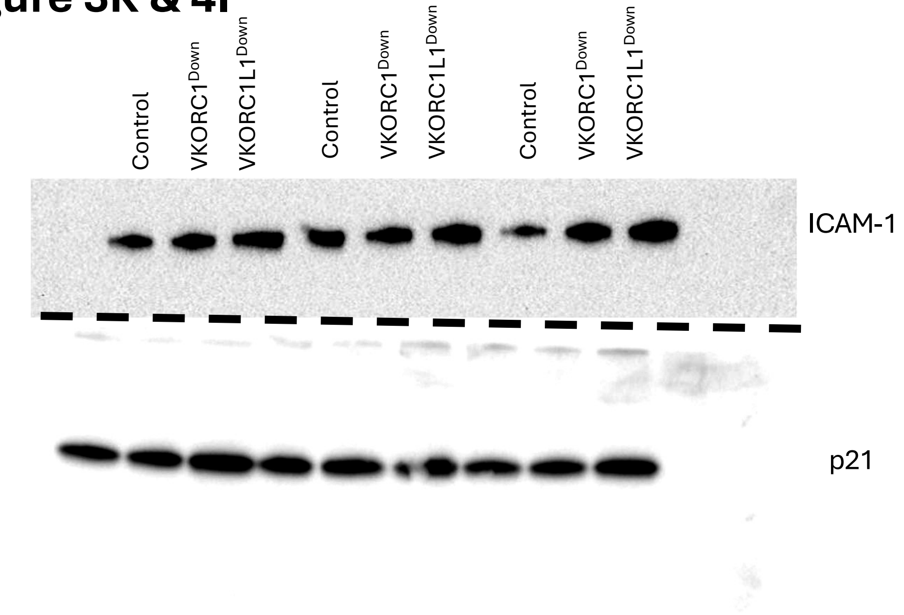

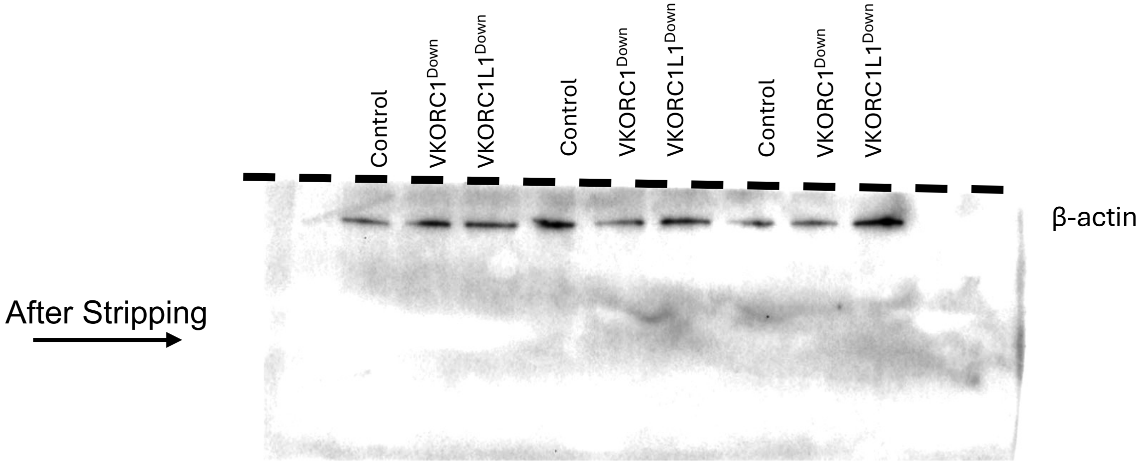

Uncropped Blots

Figure S6B

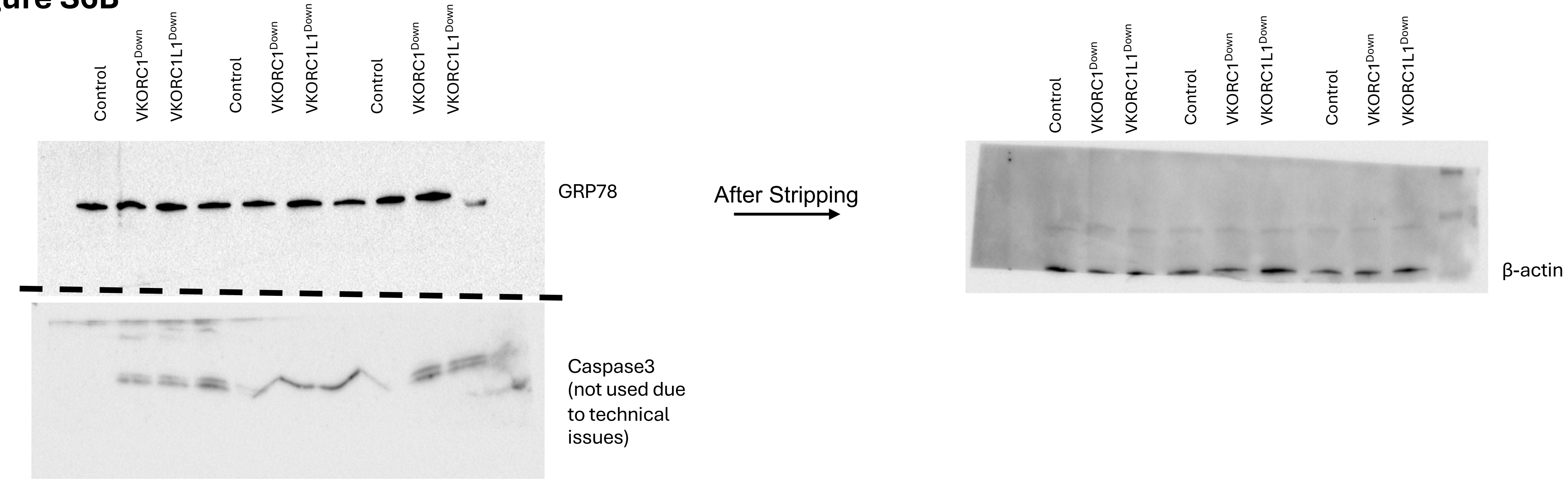

Supplement: Supplementary file 2 — Supplementary Material 2 [file 41598_2026_54463_MOESM2_ESM.pdf]
