# Supplementary material for: VKORC1L1–mediated vitamin K recycling counters ferroptosis to promote endothelial repair
Source: Sci Rep. 2026 Jun 19;16:19171. doi: 10.1038/s41598-026-54463-7 (PMC13282403; doi:10.1038/s41598-026-54463-7)
Supplement: Supplementary file 3 — Supplementary Material 3 [file 41598_2026_54463_MOESM3_ESM.pdf]

A

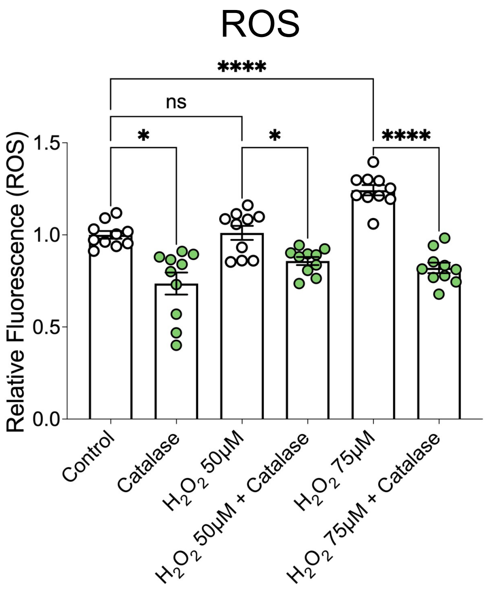
ROS
****
ns
*
*
****
Relative Fluorescence (ROS)
1.5
1.0
0.5
0.0
Control
Catalase
H_2O_2 50µM
H_2O_2 50µM + Catalase
H_2O_2 75µM
H_2O_2 75µM + Catalase

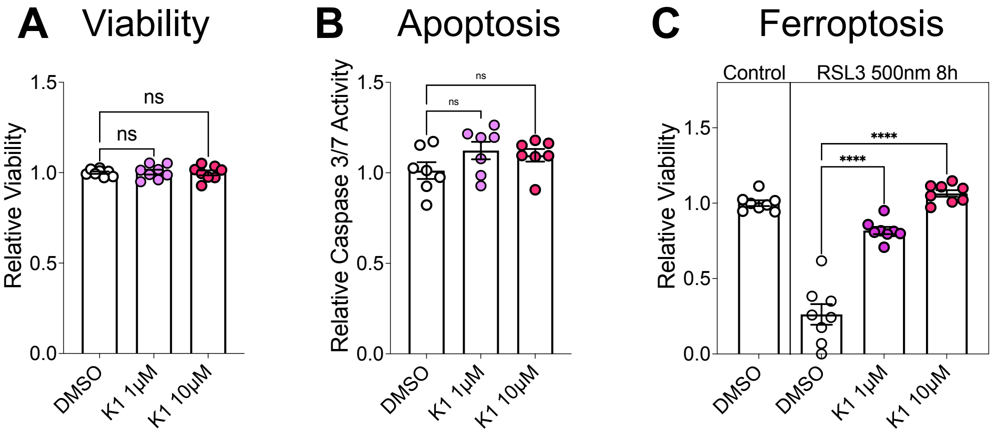
A Viability
B Apoptosis
C Ferroptosis
Relative Viability
1.5
1.0
0.5
0.0
ns
ns
DMSO
K1 1μM
K1 10μM
Relative Caspase 3/7 Activity
ns
ns
Control
RSL3 500nm 8h
****
****

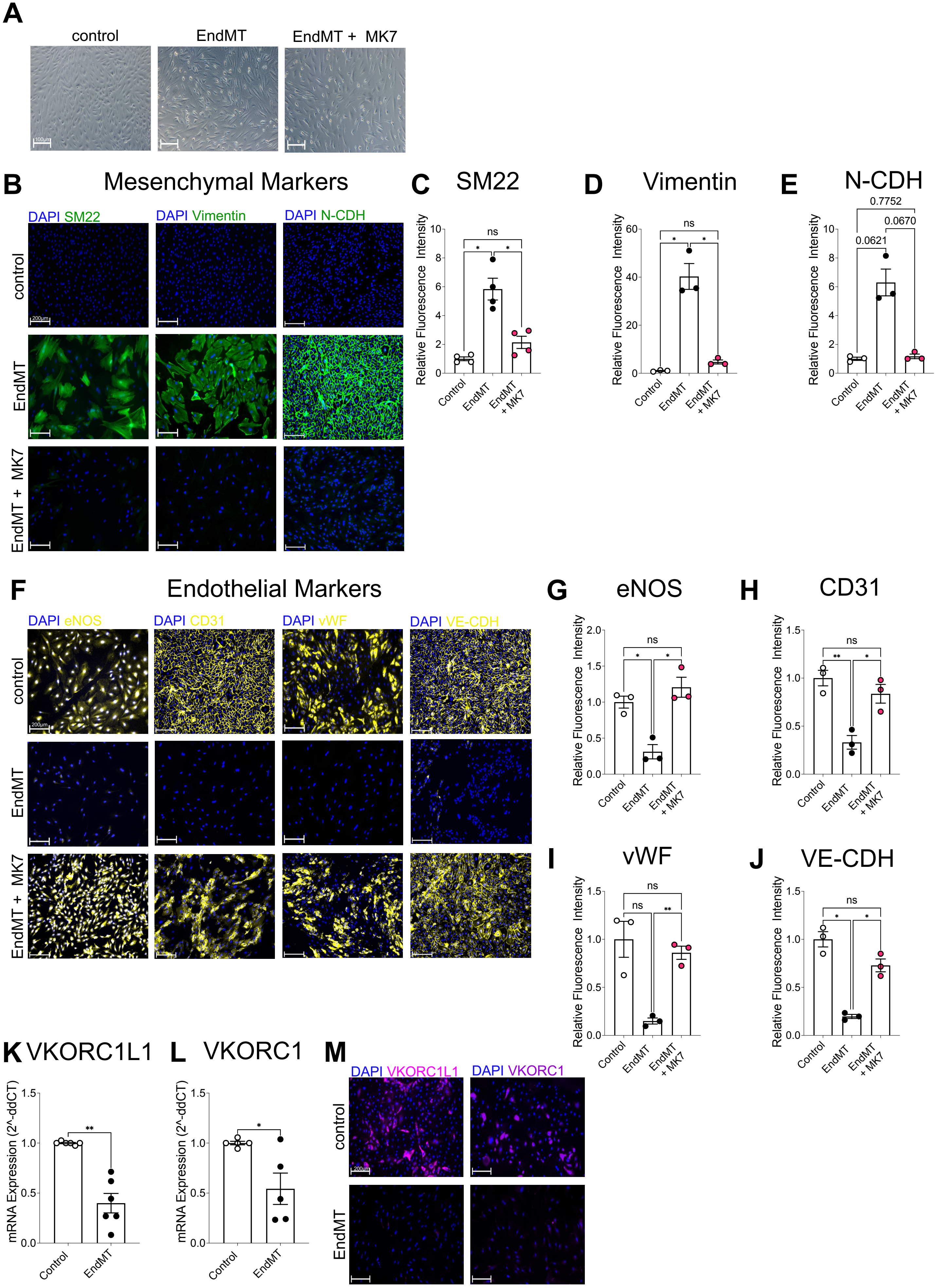
A
control
EndMT
EndMT + MK7
100μm
B
Mesenchymal Markers
DAPI SM22
DAPI Vimentin
DAPI N-CDH
control
EndMT
EndMT + MK7
200μm
C
SM22
D
Vimentin
E
N-CDH
Relative Fluorescence Intensity
ns
*
0.7752
0.0670
0.0621
Control
EndMT
EndMT + MK7
F
Endothelial Markers
DAPI eNOS
DAPI CD31
DAPI vWF
DAPI VE-CDH
control
EndMT
EndMT + MK7
200μm
G
eNOS
H
CD31
I
vWF
J
VE-CDH
Relative Fluorescence Intensity
ns
*
**
Control
EndMT
EndMT + MK7
K
VKORC1L1
L
VKORC1
mRNA Expression (2^-ddCT)
**
*
Control
EndMT
M
DAPI VKORC1L1
DAPI VKORC1
control
EndMT
200μm

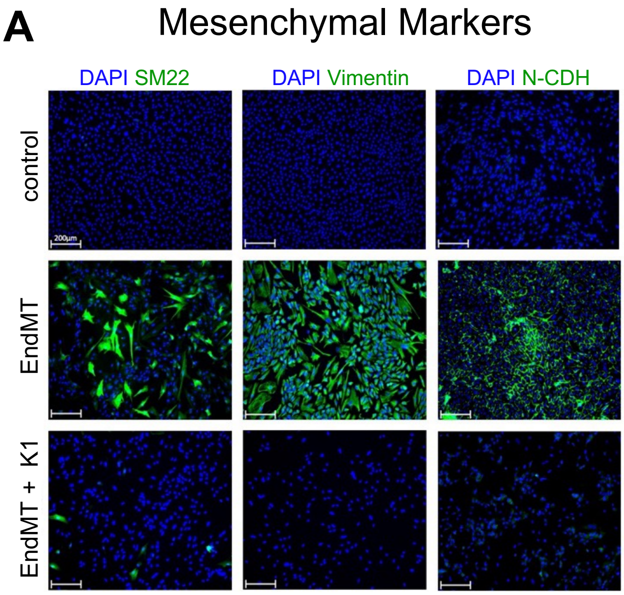
A
Mesenchymal Markers
DAPI SM22
DAPI Vimentin
DAPI N-CDH
control
EndMT
EndMT + K1
200µm

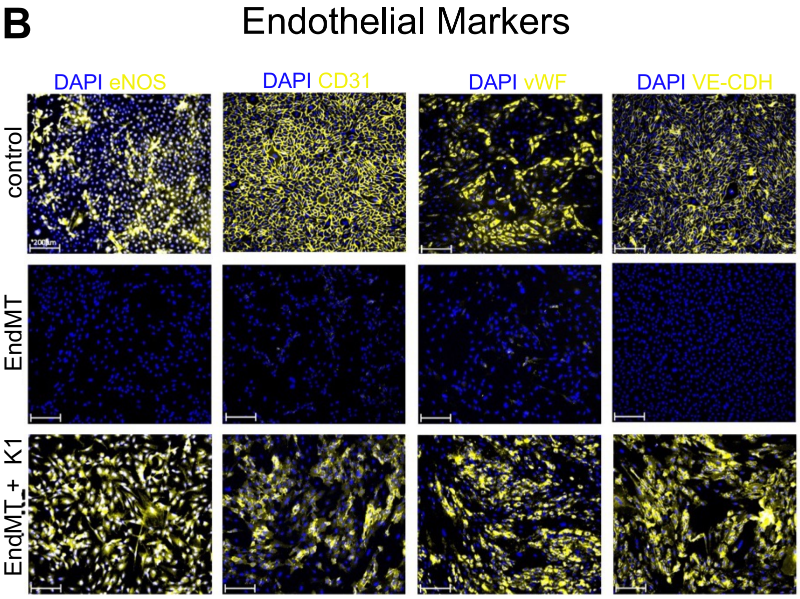
B
Endothelial Markers
DAPI eNOS
DAPI CD31
DAPI vWF
DAPI VE-CDH
control
EndMT
EndMT + K1
200µm

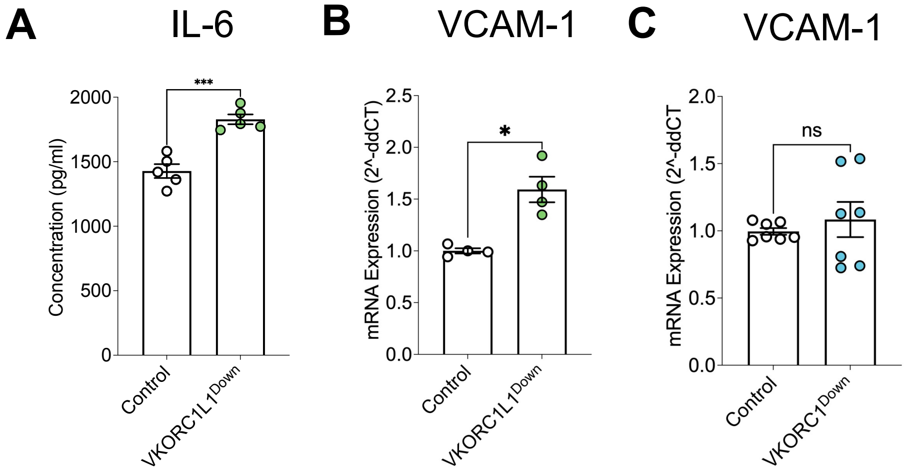
A
IL-6
Concentration (pg/ml)
2000
1500
1000
500
0
***
Control
VKORC1L1Down
B
VCAM-1
mRNA Expression (2^-ddCT)
2.5
2.0
1.5
1.0
0.5
0.0
*
Control
VKORC1L1Down
C
VCAM-1
mRNA Expression (2^-ddCT
2.0
1.5
1.0
0.5
0.0
ns
Control
VKORC1Down

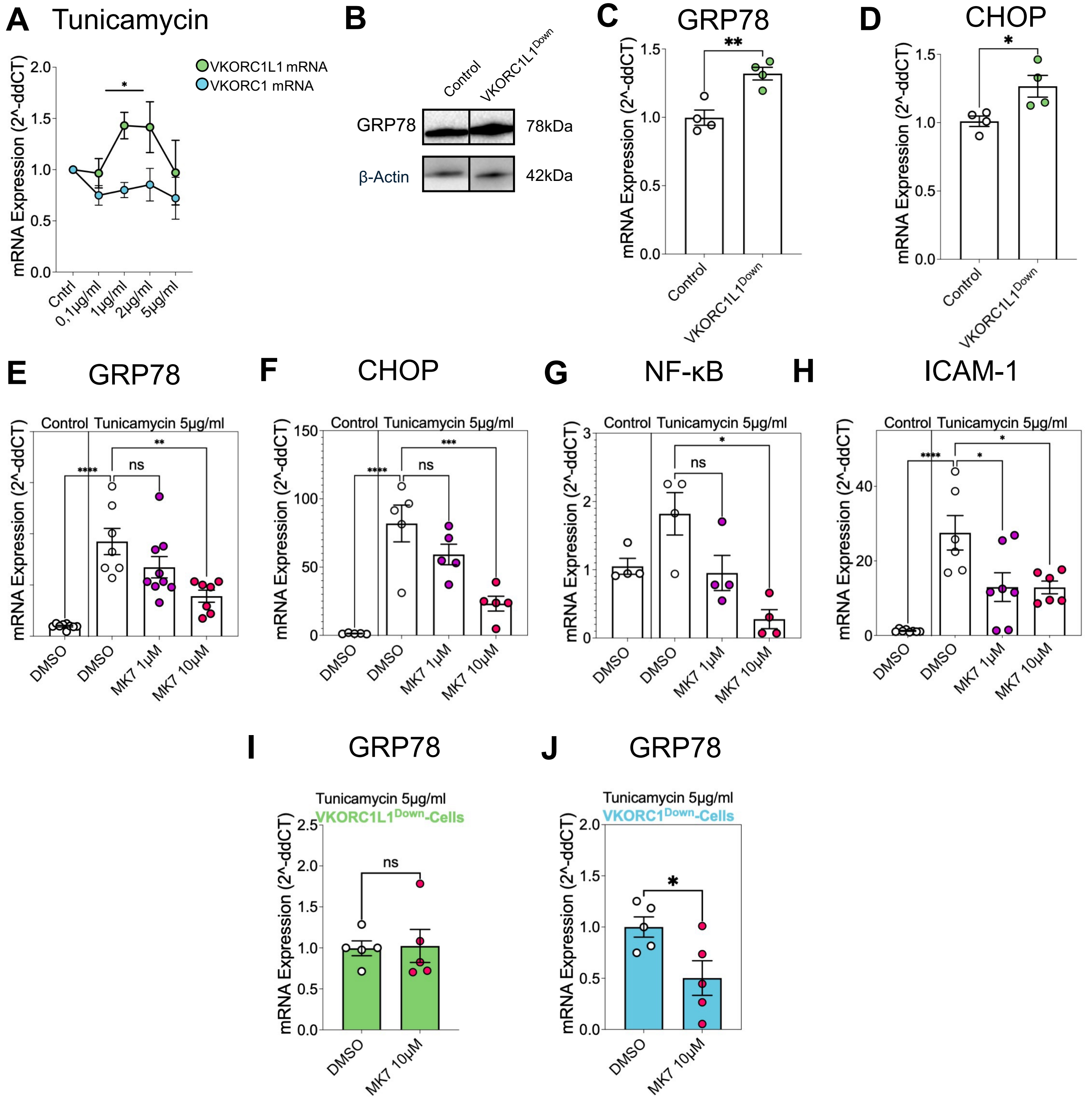
A
Tunicamycin
VKORC1L1 mRNA
VKORC1 mRNA
mRNA Expression (2^-ddCT)
Cntrl
0,1µg/ml
1µg/ml
2µg/ml
5µg/ml
B
Control
VKORC1L1Down
GRP78
78kDa
β-Actin
42kDa
C
GRP78
D
CHOP
Control
VKORC1L1Down
E
GRP78
F
CHOP
G
NF-κB
H
ICAM-1
Control
Tunicamycin 5µg/ml
ns
DMSO
MK7 1µM
MK7 10µM
I
GRP78
Tunicamycin 5µg/ml
VKORC1L1Down-Cells
J
GRP78
Tunicamycin 5µg/ml
VKORC1Down-Cells
