# Supplementary material for: VKORC1L1–mediated vitamin K recycling counters ferroptosis to promote endothelial repair
Source: Sci Rep. 2026 Jun 19;16:19171. doi: 10.1038/s41598-026-54463-7 (PMC13282403; doi:10.1038/s41598-026-54463-7)
Supplement: Supplementary file 4 — Supplementary Material 4 [file 41598_2026_54463_MOESM4_ESM.docx]

**Figure Legends**

**Figure 1 Validation of ROS-Assay**

Relative Fluorescence Intensity depicting cellular ROS levels (measured by DCFDA-assay) in HCAEC treated with Catalase (1000 U/ml, 30 min pre-treatment before H2O2 treatment), H2O2 (50 or 75 µM, 45 min) or combination of both. Cells were then stained with DCFDA (50 µM for 45 min at 37°C in the dark) and fluorescence was read. This validation experiment demonstrated a significant increase in ROS levels upon stimulation with 75 µM of H2O2. Catalase attenuated H₂O₂-induced ROS generation in HCAEC. N = 5. Data are shown as mean ± SEM

**Figure 2 Vitamin K1 protects against ferroptosis**

**A–B**Cell viability (AlamarBlue) and apoptosis (caspase-3/7) in HCAEC after 24 h K1 (1–10 µM); n = 8 (A) and 7 (B). **C** Viability after ferroptosis induction with RSL3 (500 nM, 8 h) ± K1; n = 8. Statistics: one-way ANOVA + Dunnett (A-C); Data are mean ± SEM. *P < 0.05; **P < 0.01; ***P < 0.001; ****P < 0.0001.

**Figure 3 Vitamin K2 (MK-7) limits endothelial-to-mesenchymal transition (EndMT)**

**A**Bright-field morphology of HCAEC cultured 96 h in EndMT medium ± MK-7 (5 µM); scale 100 µm; n = 3. **B–E**Immunofluorescence (left) and per-cell quantification of mesenchymal markers after 96 h EndMT ± MK-7: SM22 (C), Vimentin (D), N-cadherin (E); scale 200 µm; n = 3–4. **F–J**Endothelial markers under the same conditions: eNOS (F), CD31 (G), vWF (H), VE-cadherin (I); scale 200 µm; n = 3. **K–L**qPCR for VKORC1L1 (I) and VKORC1 (J) following 96 h EndMT; n = 6 (K) and 5 (L). **M**VKORC1L1 (left) and VKORC1 (right) immunofluorescence after EndMT; scale 200 µm; n = 3. Statistics: one-way ANOVA + Dunnett for B–J; unpaired two-tailed t-test for J–K Data are mean ± SEM; *P < 0.05, **P < 0.01, ***P < 0.001, ****P < 0.0001.

**Figure 4 Vitamin K1 mirrors MK-7 in inhibiting EndMT**

**A** Immunofluorescence of mesenchymal markers after 96 h EndMT ± K1: SM22, Vimentin, N-cadherin. scale 200 µm; n = 3–4. **B**Immunofluorescence of endothelial markers after 96 h EndMT ± K1: eNOS, CD31, vWF, VE-Cadherin. scale 200 µm; n = 3–4.

**Figure 5 Vascular Inflammation upon VKOR-Enzyme knockdown**

**A** IL-6 release (ELISA) 24 h after VKORC1L1 knock-down; n = 5. Statistics: unpaired t-test (all panels); **B–C**qPCR of VCAM 24h following knockdown of VKORC1L1 (B, n = 4) or VKORC1 (C, n = 6). mean ± SEM; *P < 0.05, **P < 0.01.

**Figure 6 ER stress links VKORC1L1 to endothelial inflammation**

**A** Dose-dependent induction of VKORC1L1 (but not VKORC1) mRNA by tunicamycin (0.1-5 µg ml⁻¹, 6 h); n = 5. **B** Representative immunoblot of GRP78 (n = 3) with β-actin loading control. **C-D** qPCR for GRP78 and CHOP up- 24 h after VKORC1L1 silencing; n = 5. **E-H**  MK-7 pre-treatment (0.1–10 µM, 24 h) attenuates tunicamycin-induced GRP78, CHOP, NF-κB and ICAM-1; n = 5-7. **I-J** MK-7 fails to suppress GRP78 when VKORC1L1 is depleted; n = 4. Statistics: one-way ANOVA + Dunnett (E-H); unpaired two-tailed t-test (C-D, I-J) or two-way ANOVA (A). Data are mean ± SEM. *P < 0.05; **P < 0.01; ***P < 0.001; ****P < 0.0001.

.
